# Detection of Ribosomal DNA Sequence Polymorphisms in the Protist *Plasmodiophora brassicae* for the Identification of Geographical Isolates

**DOI:** 10.3390/ijms18010084

**Published:** 2017-01-04

**Authors:** Rawnak Laila, Arif Hasan Khan Robin, Kiwoung Yang, Gyung Ja Choi, Jong-In Park, Ill-Sup Nou

**Affiliations:** 1 Department of Horticulture, Sunchon National University, Suncheon 540-950, Korea; rawnak.2010@gmail.com (R.L.); gpb21bau@gmail.com (A.H.K.R.); ykw7685@naver.com (K.Y.); jipark@sunchon.ac.kr (J.-I.P.); 2 Center for Eco-friendly New Materials, Korea Research Institute of Chemical Technology, Daejeon 34114, Korea; kjchoi@krict.re.kr

**Keywords:** *Plasmodiophora brassicae*, ribosomal DNA, geographical isolates, sequence variation, intraspecific polymorphism, single-nucleotide polymorphism

## Abstract

Clubroot is a soil-borne disease caused by the protist *Plasmodiophora brassicae* (*P. brassicae*). It is one of the most economically important diseases of *Brassica rapa* and other cruciferous crops as it can cause remarkable yield reductions. Understanding *P. brassicae* genetics, and developing efficient molecular markers, is essential for effective detection of harmful races of this pathogen. Samples from 11 Korean field populations of *P. brassicae* (geographic isolates), collected from nine different locations in South Korea, were used in this study. Genomic DNA was extracted from the clubroot-infected samples to sequence the ribosomal DNA. Primers and probes for *P. brassicae* were designed using a ribosomal DNA gene sequence from a Japanese strain available in GenBank (accession number AB526843; isolate NGY). The nuclear ribosomal DNA (rDNA) sequence of *P. brassicae*, comprising 6932 base pairs (bp), was cloned and sequenced and found to include the small subunits (SSUs) and a large subunit (LSU), internal transcribed spacers (ITS1 and ITS2), and a 5.8s. Sequence variation was observed in both the SSU and LSU. Four markers showed useful differences in high-resolution melting analysis to identify nucleotide polymorphisms including single- nucleotide polymorphisms (SNPs), oligonucleotide polymorphisms, and insertions/deletions (InDels). A combination of three markers was able to distinguish the geographical isolates into two groups.

## 1. Introduction

Clubroot is a soil-borne disease caused by the protist, *Plasmodiophora brassicae* (*P. brassicae*) [1]. It is an economically devastating disease in *Brassica rapa* and other cruciferous crops worldwide. Globally, it infects around six million hectares of *Brassica* crops, accounting for around 11% of *Brassica* crop production [2,3,4]. In China in 2008, 17% of young plants and 15% of mature *Brassica* plants in the field were infected, which ultimately resulted in a 10.2% yield loss [5]. In Korea, clubroot has recently become a severe, year-round threat to the production and breeding of Chinese cabbage, which is the most important ingredient of the famous Korean dish *baechu kimchi*. The first evidence of clubroot caused by *P. brassicae* was reported in Russia in 1878 by Woronin, as cited by Karling in 1968 [6]. In England, the disease was widely reported by the end of the 18th century as observed from European herbal products. Clubroot disease was reported in Japan as early as the 1890s and in Korea in the 1920s [7]. 

*P. brassicae* has a complicated life cycle consisting of three stages: survival in the soil as inactive spores, initial infection of root hairs by forming primary zoospores, and secondary infection of the cortex by forming secondary zoospores [8,9]. Infection with *P. brassicae* leads to abnormal tissue proliferation on the roots of susceptible plants, producing galls referred to as “clubs” [10,11]. This abnormal growth inhibits the uptake of water and nutrients by the roots, slowing the growth of the aboveground plant tissues and reducing the quality and commercial value of the plant products [4,12]. The *P. brassicae* pathogen forms resting spores that are released from crumbling galls and have the potential to survive in the soil for 10–20 years [13], making the disease difficult to eradicate. In a non-rotational cropping system, these resting spores multiply exponentially, leading to increased disease severity in successive crops.

*P. brassicae* belongs to the phylum Cercozoa in the kingdom Rhizaria, the class Plasmodiophoromycetes, and the order Plasmodiophorales [14,15]. Information on the genomic structure of *P. brassicae* is very limited since it is an obligate intracellular parasite and difficult to culture in the laboratory. The characterization of a pathogen in plant tissues is lengthy, labor-intensive, and is complicated by environmental variations. Different pathotypes of *P. brassicae* exist in different hosts [16,17,18,19,20]. Therefore, it is important to study the genomic structure of isolates of this pathogen collected from different locations.

In plant disease diagnostics, the application of marker-based detection of isolates of a pathogen has become essential [21]. Polymerase chain reaction (PCR)-marker-based detection is advantageous for accurate identification of non-culturable obligate parasites [22]. PCR-based techniques for detection of *P. brassicae* have targeted both a single-copy sequence [22,23] and the multicopy ribosomal DNA [24,25]. Ribosomal DNA (rDNA) is a nuclear DNA sequence that codes for ribosomal RNA (rRNA), which is necessary for the synthesis of proteins, cellular growth, and development of organisms. About 80% of the rRNA present in a growing cell takes part in modulating approximately half of all cellular translation [26,27]. An rDNA repeat unit in eukaryotes generally consists of several coding regions including a smaller subunit, a larger subunit, a comparatively larger intergenic spacer (IGS), and two comparatively larger internal transcribed spacers, ITS1 and ITS2, on either side of a 5.8s subunit [28,29]. Ribosomes translate mRNA molecules to produce proteins [26,30].

**Figure 1:**
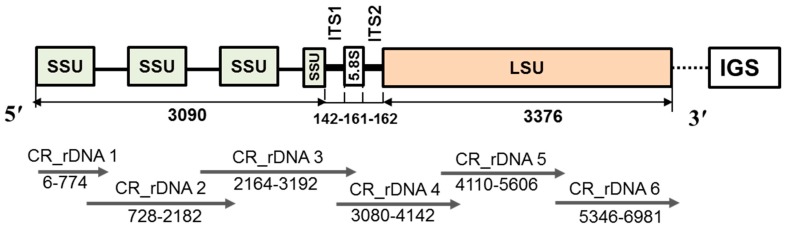
Schematic structure of the ribosomal DNA (rDNA) of *P. brassicae.* SSU, small subunit; LSU, large subunit; ITS, internal transcribed spacer; IGS, intergenic spacer based on literature [31,32]; CR_DNA (from 1 to 6) indicates marker sequences developed for cloning the target regions. Marker sequences are given in Table 1. Numbers in the figure are lengths in base pairs (bp) of each subunit cloned and sequenced in this study. The dotted line in-between LSU and IGS at the 3′ end indicates uncompleted sequences. Arrows from 5′ to 3′direction indicate length of primers.

As shown in Figure 1, the rDNA of *P. brassicae* consists of a small subunit (SSU), ITS1, 5.8s, ITS2 and a large subunit (LSU). Designing, testing, and confirming molecular markers that can quickly identify *P. brassicae* isolates collected from different geographical locations may improve efforts to halt the dissemination and better control this pathogen. *P. brassicae* isolates with geographical variation (Swedish versus UK) had single-nucleotide polymorphisms (SNP) in the ITS and 18s smaller subunits [22]. Such variation can be exploited for accurate detection of a particular isolate with SNP markers. To design these markers, molecular characterization of the rDNA is essential. Niwa et al. [31] published the complete rDNA structure of *P. brassicae* of Japanese origin (accession number AB526843; clone NGY; 9513 base pairs (bp)). The authors also sequenced partial rRNA sequences of the LSU from 25 clones from eight Japanese geographical isolates (field populations); these sequences were between 1131 and 1479 bp, and were quite diverse in nucleotide sequence compared to the NGY isolate. Recently, Schwelm et al. [32] published nearly complete rDNA sequences of two isolates of *P. brassicae* of European origin, AT (KX011115, 7909 bp) and e3 (KX0111135, 7382 bp), which were nearly identical to each other at their 28s LSU. These authors also published partial sequences for 28s rRNA (1143 bp) from 19 other *P. brassicae* isolates originating from Germany (KX011116), Sweden (KX011117–KX011123), New Zealand (KX011124), Canada (KX011125–KX011132), and South Korea (KX011133–KX011134). These 19 partial rDNA sequences originating from five different countries were identical to AT and e3 isolates originating from Germany and Austria, respectively [32]. In Korea, a number of geographical isolates of *P. brassicae* have been collected [33]. The aim of the present study was to determine the complete structure of the rDNA repeat unit of *P. brassicae* and identify the polymorphisms among Korean field populations of different geographic origin in order to develop molecular markers for characterization of the isolates.

## 2. Results

### 2.1. P. brassicae DNA Isolation 

We initially tried several previously used approaches to extract DNA from clubroot-infected *B. rapa* samples, including boiling [22,34], DNA miniprep kit extraction [35,36], and Percoll gradient separation [31]. We found that the EZNA SP Fungal DNA Miniprep Kit protocol (Omega Bio-Tek, Inc., Norcross, GA, USA) yielded comparatively better quality *P. brassicae* DNA (Figure S1). DNA samples prepared using the EZNA SP Fungal DNA Miniprep Kit did not give rise to products of the target size using *B. rapa*-specific primers (Figure S1), confirming its suitability for preparation of *P. brassicae* DNA to clone its rDNA. Accordingly, we used DNA prepared in this manner for all further analyses.

### 2.2. Nucleotide Sequence in ITS1 and ITS2 was Conserved

**Figure 2:**
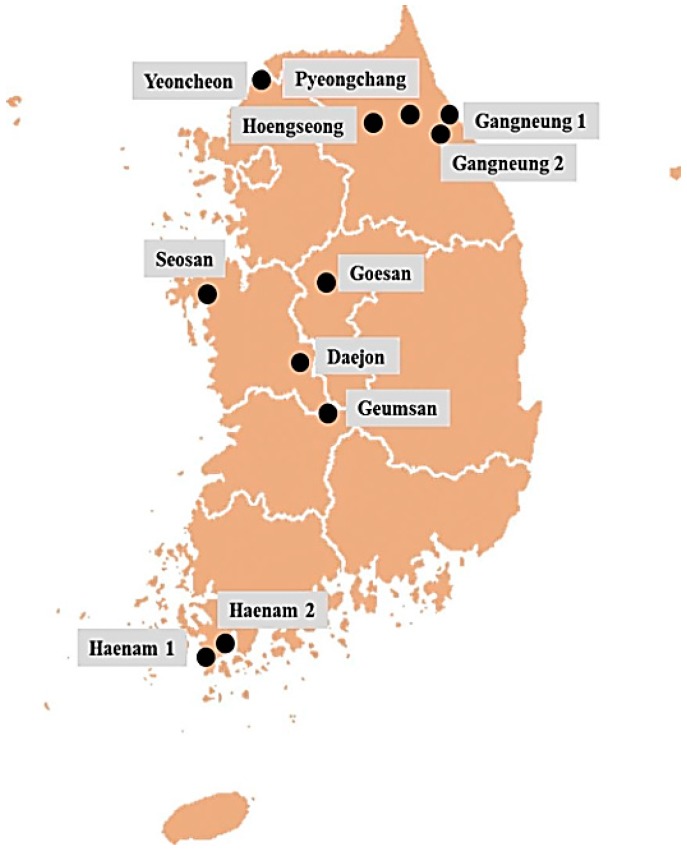
Sampling sites of 11 *P. brassicae* field populations (geographical isolates) from nine different regions of South Korea.

We sequenced the ribosomal DNA sequences of 11 *P. brassicae* isolates collected from nine locations in South Korea (Figure 2). The rDNA sequences consisted of 6932 bp and included a complete small subunit, 5.8s, ITS1, ITS2, and a large subunit of 3376 bp. ITS1 and ITS2 were found generally conserved in all 11 *P. brassicae* isolates, but a single-nucleotide polymorphism was detected in both spacers (Figure S2). The 5.8s region was completely conserved among 11 *P. brassicae* isolates (Figure S2).

### 2.3. Sequence Variation in the Small Subunit of the Ribosomal DNA of P. brassicae

**Figure 3:**
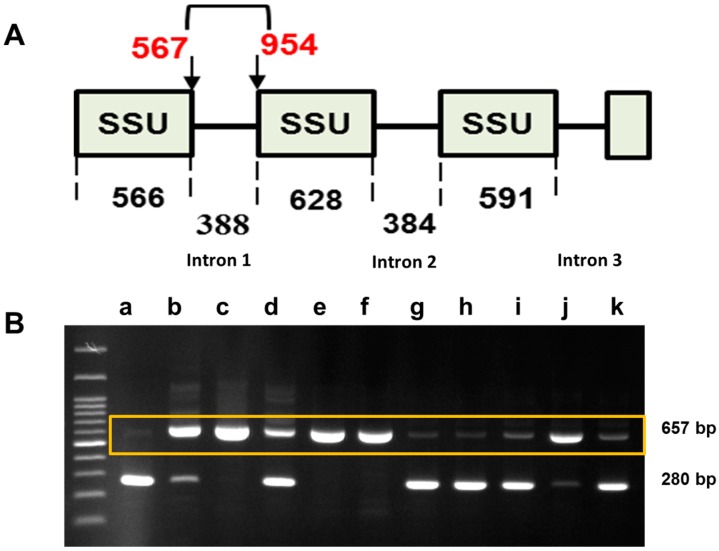
Variability in intron 1 of the small subunit (SSU) of the ribosomal DNA of *P. brassicae*. Numbers indicate the size of the SSUs and intron positions. Numbers in the figure are lengths in bp of each subunit and intron. (**A**) Schematic structure of the SSUs; red letters indicate the position of intron 1; (**B**) A deletion (388 bp; Figure S3) was detected in the a, g, h, i, and k isolates. a, Gangneung1; b, Yeoncheon; c, Daejon; d, Haenam2; e, Seosan; f, Pyeongchang; g, Gangneung2; h, Haenam1; i, Hoengseong; j, Geumsan; and k, Goesan. Primers were designed based on a reference sequence (NCBI Acc. No. U18981). The forward and reverse primer sequences were 5′-GGACTGGTAATTGGAATGAGA-3′ and 5′-TCACAGTAAACGATCAACCG-3′, respectively. The yellow box indicates the presence of 657 bp bands in the isolates.

A deletion of 388 bp in the sequences of eight isolates including Gangneung1, Gangneung2, Haenam1, Haenam2, Yeoncheon, Hoengseong, Geumsan and Goesan was detected in intron 1 region of SSU in addition to the expected product of 657 bp (Figure 3 and Figure S3) [37]. No variants were detected in this region in the following three isolates: Daejon, Seosan and Pyeongchang (Figure 3). The sequences of intron 1 of the SSU confirmed this deletion in eight isolates (Figure S3).

### 2.4. Sequence Variation in the Large Subunit of the Ribosomal DNA of P. brassicae

**Figure 4:**
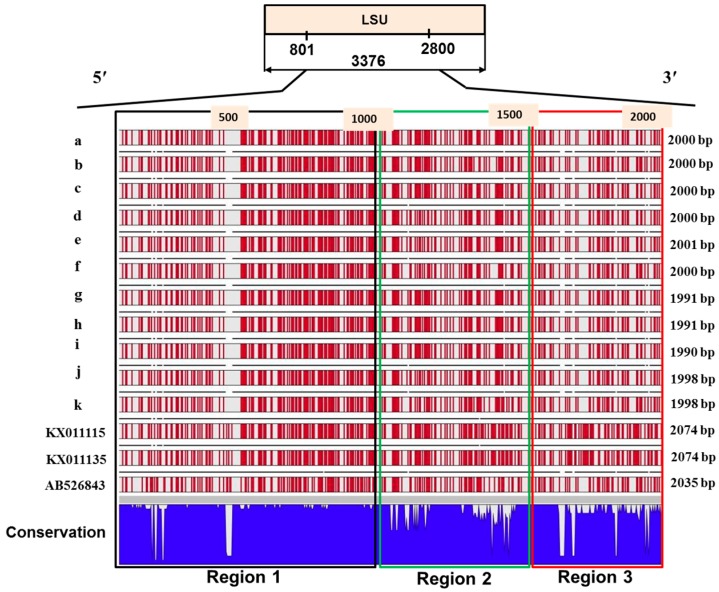
Variations in the sequences of the large subunit (between 801–2800 bp) in the 11 Korean geographical isolates of *P. brassicae,* the Japanese isolate AB526843 and two European isolates KX011115 and KX011135. Red bars and white spaces (expect gaps in annotations) represent the locations of GC and AT bases, respectively. Gaps in annotation indicate insertion-deletion (InDel) in sequences compared to consensus sequence. White gaps in conservation indicate sequence dissimilarity among isolates. a, Gangneung1; b, Yeoncheon; c, Daejon; d, Haenam2; e, Seosan; f, Pyeongchang; g, Gangneung2; h, Haenam1; i, Hoengseong; j, Geumsan; and k, Goesan.

When the sequences of the large subunit of the 11 geographical isolates were compared along with the reference sequence AB526843 and European sequences KX011115 and KX011135, at least three variable regions were identified (Figure 4 and Figure S4). No sequence variation was observed among 11 Korean field isolates and published sequences for the first 870 sequences of LSU (Figure S4), i.e., including SSU, ITS and 5.8s about 4425 bp rDNA sequences of 11 Korean field isolates were identical with three published sequences AB526843, KX011115 and KX011135. In region 1 (for the sequences between 4426 and 5421 bp), the 11 Korean geographical isolates and two European sequences KX011115 and KX011135 were strikingly different from the Japanese isolate AB526843 (Figure 4 and Figure S4). In this region, the Korean isolates were almost identical to European sequences, except that there was a 33 bp insertions/deletion (InDel) between 4788 and 4814 bp (Figure 4 and Figure S4). In region 2 (sequences between 5422 and 5585), the Korean isolates Gangneung1 (a), Yeoncheon (b), Daejon (c), Haenam2 (d), Seosan (e), and Pyeongchang (f) had nearly identical nucleotide sequences but were quite different from the other five Korean isolates: Gangneung2 (g), Haenam1 (h), Hoengseong (i), Geumsan (j), and Goesan (k) (Figure 4). The latter five isolates had greater similarity to the three published sequences in this region. In region 2, the sequences of Korean isolates between 5586 and 5747 bp were largely similar to all three published sequences (Figure 4 and Figure S4). In the 3′-end of region 2 (sequences between 5748 and 5901 bp), the Korean isolates Yeoncheon (b), Geumsan (j), and Goesan (k) showed sequence dissimilarity to the other eight Korean isolates (Figure 4). All Korean isolates showed large sequence dissimilarity with two European sequences KX011115 and KX011135 for the sequences between 5748 and 5901 bp (Figure S4). Region 2 was the most variable region where a large number of variation in A, T, G, C bases were observed among 11 Korean *P. brassicae* isolates (Table S1). Percentage G (%G) was the highest compared to the other three bases in this region (Table S1). Haenam1 and Hoengseong accounted for the highest 54.1% GC bases and the isolates Geumsan and Goesan accounted for the lowest, 49.9% GC (Table S1). In region 3, the sequence between 6081 and 7010 bp, 11 Korean isolates were almost completely dissimilar to the two European sequences KX011115 and KX011135, whereas these sequences were largely similar to the Japanese reference sequence AB526843 (Figure S4). In region 3, the Korean isolate Goesan (k) was quite similar to the Japanese isolate (Figure 4), while Yeoncheon (b) and Geumsan (j) were quite different from the other Korean isolates (Figure 4).

### 2.5. Variations in the Predicted Secondary Structures of the Variable Regions of the Large Subunit 

**Figure 5:**
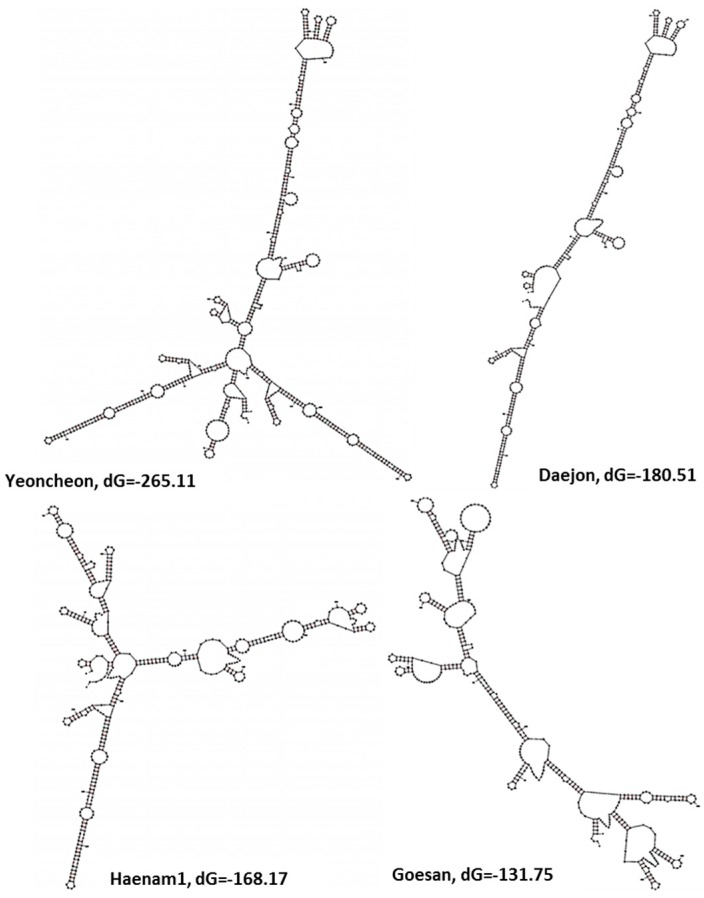
Predicted secondary structures of the most variable region of the LSU of ribosomal DNA of *P. brassicae*. dG estimates predicted free energy of folded rDNA in kcal·mol^−1^.

The prediction of secondary structure reports the available free energy, which reflects the level of activity of that rDNA in protein synthesis. The free energies are calculated as Gibbs free energy dG, a thermodynamic potential. The smaller the dG value, the higher the activity of rDNA. The secondary structure of the rDNA of the most variable regions of the large subunit between 5370 bp and 5840 bp (region 2) was predicted for all 11 Korean field isolates and the Japanese reference sequence AB526843 (Figure 5 and Figure S5). The Korean isolate Yeoncheon had the smallest predicted dG value for region 2, −265.11 kcal·mol^−1^ (Figure 5). The Korean isolates Daejon, Gangneung1, and Gangneung2 had −180 kcal·mol^−1^ or more negative free energies for that region (Figure 5 and Figure S5). Haenam1, Haenam2, Seosan, Pyeongchang, Hoengseong, and the Japanese reference isolate AB526843 had dG values between −163 and −169 kcal·mol^−1^ (Figure 5 and Figure S5). The other two isolates, Geumsan and Goesan, which were quite different from the other isolates at the 3′ end of region 2, had the highest dG values (Figure 5 and Figure S5). The isolate Yeoncheon, which had the smallest dG value, had the highest number of loops (28) compared to the other isolates in their predicted secondary structure (Figure 5).

### 2.6. Phylogenetic Analysis

**Figure 6:**
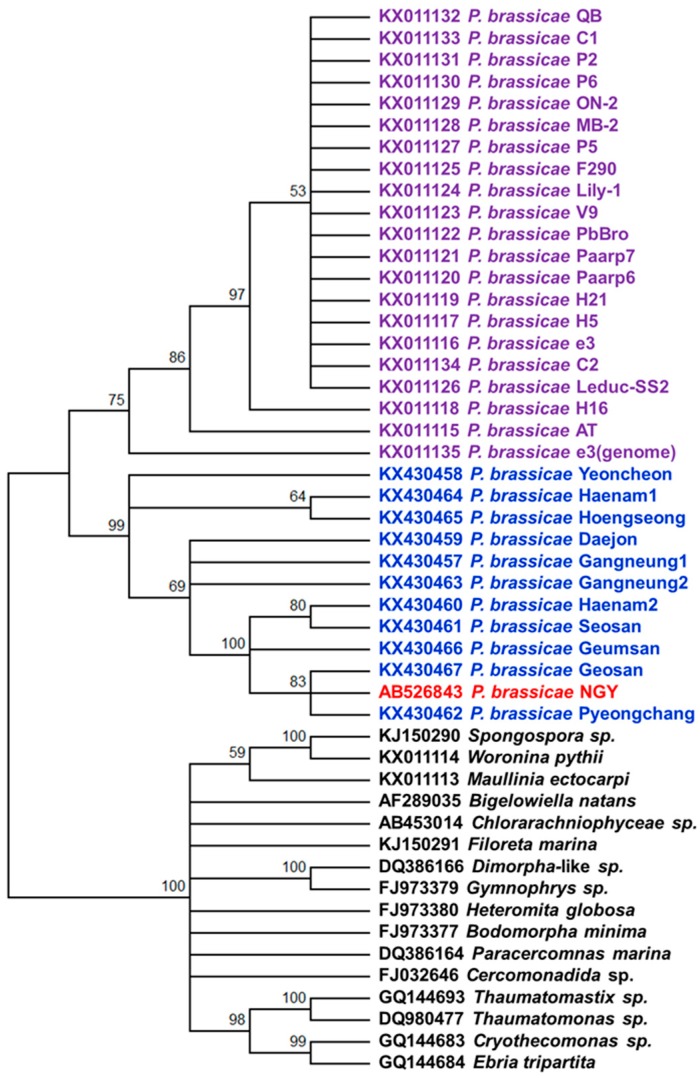
Phylogenetic tree constructed following the Maximum Likelihood method and Jukes-Cantor model in Mega6.06 software between the 11 Korean field isolates and the reference isolates of *P. brassicae*, based on variations in the nucleotide sequences of rDNA. Complete rDNA, if available, or LSU sequences were used. Accessions with “KX4304” were obtained in this study. Purple, red and blue colours indicate *P. brassicae* rDNA sequences obtained from Schwelm et al. [32], Niwa et al. [31] and this study. Black colours indicate rDNA sequences from other cercozoa species.

Two separate clusters were formed in the phylogenetic tree based on the rDNA sequences that separated *P. brassicae* isolates from other cercozoa species according to the Maximum Likelihood method (Figure 6 and Figure S6). Similar phylogenetic separation was also found in a circular cladogram constructed following the unweighted pair-group method with arithmetic mean (UPGMA) method and Kimura 80 nucleotide distance measure method (Figure S6). The 11 Korean isolates and three published sequences AB526843, KX011115 and KX011135 were in the same cluster (Figure 6 and Figure S6). However, all 11 Korean isolates formed a separate sub-cluster with the Japanese NGY isolate (Figure 6). The Korean isolates having higher dG values between −131 and −163 kcal·mol^−1^ in their predicted secondary structures, due to sequence variations in region 2 (Geosan, Geumsan, Seosan, Haenam2, and Pyeongchang), further sub-clustered together. The other six Korean isolates with smaller than −168 kcal·mol^−1^ dG clustered together, indicating that the variation in region 2 accounted for the majority of variation in phylogenetic clustering (Figure 6). The Japanese reference isolate had a closer sequence similarity with the Korean isolate Geosan that predicted the largest dG value (Figure 6). In the neighbour-joining tree, two Korean isolates, Haenam1 and Hoengseong, were closely clustered with the European sequences KX011115 and KX011135 (Figure S6).

### 2.7. Nucleotide Polymorphisms in the Ribosomal DNA of P. brassicae

**Figure 7:**
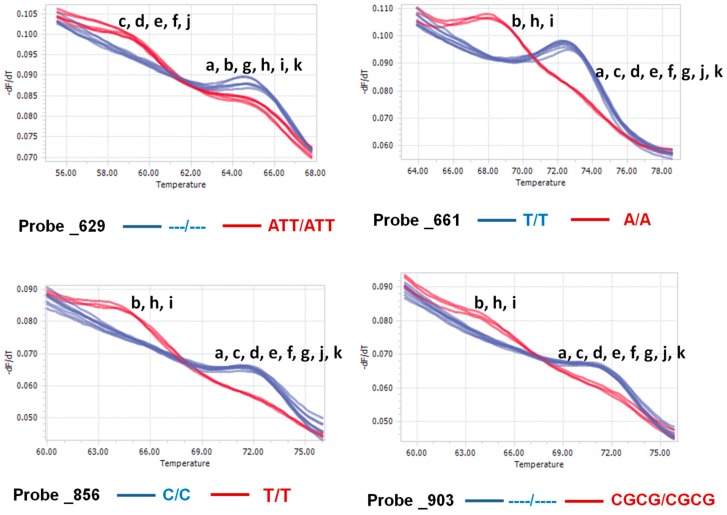
Variation due to polymorphism between Korean *P. brassicae* isolates. The melting temperatures are shown for probes (single-nucleotide and oligonucleotide polymorphism and InDel) after high-resolution melting analysis in a Roche light cycler. a, Gangneung1; b, Yeoncheon; c, Daejon; d, Haenam2; e, Seosan; f, Pyeongchang; g, Gangneung2; h, Haenam1; i, Hoengseong; j, Geumsan; and k, Goesan.

A notable number of single-nucleotide polymorphisms (SNPs), oligonucleotide olymorphisms, and insertions-deletions (InDels) among the 11 Korean field isolates were observed. Such variation was predominant in the large subunit (Figure 4). To develop specific markers based on single-nucleotide or oligonucleotide polymorphisms, ten probes were designed and tested through high-resolution melting. Four out of the ten probes resulted in significant melting temperature differences (Figure 7); however, the remaining six probes showed no differences (Figure S7). Probes 661, 856 and 903 clearly separated Yeoncheon, Haenam1, and Hoengseong (red peaks) from the other eight isolates (Figure 7). The melting temperature difference between the two groups for probes 661, 856 and 903 were 5, 8 and 7 °C, respectively (Figure 7). Probes 661 and 856 separated the Korean isolates based on SNPs, whereas probe 903 separated the two groups based on an InDel of four nucleotides. Probe 629 had about a 6 °C melting temperature difference between peaks produced by Daejon, Haenam2, Seosan, Pyeongchang, Geumsan (red peaks, Figure 7), and the other six isolates (Gangneung1, Yeoncheon, Gangneung2, Haenam1, Hoengseong, Goesan; blue peaks in Figure 7) due to variation in three adjacent nucleotides. 

## 3. Discussion

### 3.1. Successful Isolation, Cloning and Sequencing of P. brassicae Ribosomal DNA 

The objective of the present study was to clone and sequence the rDNA of the protist *P. brassicae* for the development of molecular markers for the detection of isolates. The isolation of this DNA, as well as the cloning and sequencing, was challenging. As *P. brassicae* is an obligate parasite, the first challenge was to obtain bands using *P. brassicae* rDNA-specific primers and also to confirm that the same primer does not amplify *B. rapa* rDNA. Here, we successfully isolated better quality *P. brassicae* DNA and confirmed that the cloned nucleotide sequences of the small and large subunits were not from the host rDNA, a task complicated by the fact that the rDNA of *P. brassicae* and Chinese cabbage had high sequence similarity (Figure S8). We used alignment of the rDNA sequences from a Japanese *P. brassica* isolate AB526843 [31] and *Brassica rapa* KM538956 [38] to determine the similarity between the two organisms and found that both sequences were 60% identical, which makes it difficult to clone rDNA of *P. brassicae*.

### 3.2. Ribosomal DNA Sequence Variation in the SSU and LSU of P. brassicae rDNA 

A large deletion of 388 base pairs at intron 1 of the SSU clearly separated the Korean field isolates of *P. brassicae* into two groups (Figure 3). Three large deletions of 388, 383 and 442 bp were previously identified in *P. brassicae* field isolates from the United States [37]. These findings suggest that variation in the SSU of the rDNA is associated with evolutionary changes that result in distinct field isolates. Variations in the SSU have similarly been reported between *Rhizophagus irregularis* and *Gigaspora margarita* arbuscular mycorrhizal fungi [39]. This variation accounted for 1%, 4% and 6% of sequence differences for the SSU, LSU, and ITS between the species, respectively. Intra-isolate nucleotide variations in the ITS, LSU, and SSU have also been reported for both species [39], in agreement with our results. The variations, which were attributed to both nucleotide polymorphisms and InDels, separated the Korean isolates into two groups based on the three probes 629, 661 and 856 (Figure 7). These variations may have arisen due to mutations in their ancestral rDNA sequence and might influence the functionality of this protist. 

Similar to our results, Niwa et al. [31] reported intra-species polymorphism in LSU of *P. brassicae* (Figure 4 and Figure S4). The authors collected 26 clones from eight Japanese geographical isolates from eight different locations of Japan. They sequenced complete LSU sequence of one isolate (NGY) and partial sequence between 1131 and 1479 bp of the remaining 25 clones. In contrast to Niwa et al. [31] and this study, Schwelm et al. [32] reported that LSU of rDNA in *P. brassicae* does not contain intra-species polymorphism. The conclusion of Schwelm et al. [32] was primarily based on two nearly complete sequences for 28s LSU from two European isolates, isolate AT from Austria and isolate e3 from Germany. The authors also compared partial sequences of 1143 bp from another 19 isolates collected from six different countries including Sweden, Canada, New Zealand and South Korea. Notably, out of those latter 1143 bp sequences, about 980 bp (from 4816 to 5746 bp and from 5902 to 5951 bp) were almost identical with the majority of the Korean isolates from this study (Figure S4). 

The variations observed in the SSU and LSU of the Korean field isolates of *P. brassicae* were another notable finding of this study. Indeed, two field isolates, Seosan and Daejon, representing different groups in the phylogenetic tree (Figure 6), produced different-sized galls in the Chinese cabbage cultivar “Bulamsam” when about 100 plants, grown in a plant culture room, were infected with each of those two different isolates (Figure S9). Field isolates of Seosan produced visible galls 28 days after infection, whereas those of Daejon produced galls one week earlier (data not presented) [33]. Such functional variability between isolates might be associated with both structural variations in the rDNA, as these two rDNAs have different available free energies in predicted secondary structures (Figure 5 and Figure S5). In addition, the isolates Daejon had higher GC% in the region 2 of LSU compared to Seosan (Table S1). Moreover, these isolates Daejon (pathotype 2) and Seosan (pathotype 4) were classified into two different pathotypic groups by Kim et al. [40]. Thus, variation in SSU and LSU sequences might be related to functional variation of *P. brassicae* field isolates. 

### 3.3. Detection of Isolates Using Functional Nucleotide Variations

Nuclear rDNA sequences are the most commonly used markers for specific detection of a variety of fungi [41,42,43]. Our probes 661, 856 and 903 clearly separated three isolates—Yeoncheon, Haenam1, and Hoengseong—into a separate cluster from the other eight isolates due to a significant melting temperature difference (Figure 7). Among these three probes, 661 produced peaks for both isolate groups with a larger difference in fluorescence value (^−dF^/_dT_) and, therefore, probe 661 could be recommended as a comparatively better high resolution melting (HRM) marker for detecting Korean *P. brassicae* isolates. These results were consistent with these three isolates having a strong evolutionary relationship (Figure 6). Our data indicate that nucleotide variations in field isolates of *P. brassicae* can be used to develop isolate-specific markers. Four separate probes were able to group the 11 field isolates into two groups. Since the four validated HRM sequences reside on the intron 1 of SSU, functionality of those markers is therefore restricted to the obligatory presence of full intron 1 sequence in SSU. In absence of intron 1 sequences, these markers would be not useful. Our next objective will be to sequence the complete genomic sequence of all 11 *P. brassicae* isolates in order to develop isolate-specific markers to distinguish all field isolates of this obligate parasite. 

## 4. Materials and Methods 

### 4.1. Collection of Field Populations

The clubroot-infected Chinese cabbage (*Brassica rapa*) samples were collected between 2009 and 2013 from nine different regions of South Korea: Gangneung (two locations), Yeoncheon, Daejon, Haenam (two locations), Seosan, Pyeongchang, Hoengseong, Geumsan, and Goesan, as described by Kim et al. [40] and Jo et al. [44] (Figure 2). The collected clubroot galls were washed thoroughly and stored at −80 °C. 

### 4.2. DNA Extraction

We used a modified EZNA SP Fungal DNA Miniprep Kit extraction kit (Omega Bio-Tek, Doraville, GA, USA) to extract DNA from the clubroot-infected samples. Before this, we tried the boiling method [22,34] the DNA miniprep kit extraction method [35,36], and the Percoll gradient separation method [31] to extract DNA from the clubroot-infected samples. About 100–130 mg of infected root tissue was collected in a 1.5-mL tube. The gall samples were washed carefully and the outer layer of each gall was peeled off. The galls were surface-sterilized with 70% (*v*/*v*) ethanol for 30 s. Each peeled gall sample was homogenized in 1.5 mL cetrimethyl ammonium bromide (CTAB) (20 g CTAB, 12.11 g Tris, 81.8 g NaCl, and 7.4 g·L^−1^ disodium-ethylene diamine tetraacetic acid (EDTA); pH 8.0) solution with 30 µL proteinase K (20 mg·L^−1^, GeneScan) and 30 µL DNaseI (D-4263, Sigma, St. Louis, MO, USA) and incubated at 60 °C for 2 h. DNase I was used to eliminate *B. rapa* host DNA [45,46,47] as it has been reported that during the infection cycle, host DNA is taken up by *P. brassicae* [48]. The suspension was then centrifuged at 10,000× *g* for 5 min and 1 mL supernatant was mixed with 400 µL SFG1 Buffer and 4 µL RNase A (Omega Bio-Tek) in a 1.5-mL tube, followed by vortexing at maximum speed to mix thoroughly. *P. brassicae* DNA was then collected following the EZNA SP Fungal DNA Miniprep Kit protocol. The concentration of the prepared DNA was measured in a Nanodrop ND-1000 spectrophotometer (NanoDrop Technologies, Wilmington, DE, USA) and stored at −20 °C.

### 4.3. PCR Amplification, Cloning and Sequencing

Primers were designed using a *P. brassicae* ribosomal DNA sequence available in GenBank, Acc. No. AB526843 [31]. Six primers were designed using Primer3 software (Available online: http://primer3.ut.ee/; Figure 1, Table 1) to clone the entire small subunit and a major portion of the large subunit of the ribosomal DNA of *P. brassicae*. PCR amplification was carried out in 50 µL volumes with the Phusion High-Fidelity DNA PCR kit (New England Biolabs, Inc., Ipswich, MA, USA) containing 1 µL gDNA, 2 µL each primer, 0.6 µL Phusion DNA Polymerase, 10 µL Phusion HF Buffer, 4 µL dNTPs, and 29.4 µL sterile distilled water. The mixtures were incubated in an Eppendrof thermal cycler (Eppendrof AG, Hamburg, Germany) for 30 cycles of 1 min at 94 °C, 1 min at 52 °C, and 3 min at 72 °C. After 30 cycles, the samples were incubated for an additional 5 min at 72 °C. The PCR products were separated on a 1.2% agarose gel with 1× TBE buffer as a single band with a 100-bp DNA ladder. DNA fragments were purified with the Promega extraction kit (Promega, Madison, WI, USA) according to the manufacturer’s instructions. Cloning was performed using the Topcloner Blunt Core kit (Enzynomics Inc., Daejeon, Korea) according to the manufacturer’s instructions. The ligated DNA was transferred into competent *E. coli* cells. The recombinant plasmids were purified using the plasmid mini kit (Burlington, ON, Canada) according to the manufacturer’s instructions. The cloned DNAs were sequenced with the universal primers M13F and M13RpUC using the ABI 3730XL sequencer (Macrogen Co., Seoul, Korea). Three clones per isolate were used for sequencing. The sequencing results were compared with the reference sequence (Accession Number AB526843) [31] to compare the length of the ribosomal DNA and the sequences of the SSU, ITS1, 5.8s, ITS2 and LSU.

### 4.4. Sequence Analysis

The sequences obtained after cloning were aligned and assembled together using CLC Main Workbench version 7. The assembled sequences of the 11 isolates were submitted to the GenBank database of NCBI with the following accession numbers: KX430457–KX430467 (Table 2). CLC Main Workbench version 7 was used to identify AT- and GC-rich regions compared to GenBank Accession Numbers AB526843, KX011115 and KX011135. GeneDoc v2.6.002 (available online: http://www.nrbsc.org/gfx/genedoc/index.html) was used for multiple sequence alignment of the rDNA sequences of the 11 Korean isolates with the reference sequence, GenBank Accession Number AB526843. The rDNA sequences of *P. brassicae* were also aligned with that of *B. rapa* using CLC Main Workbench version 7. Molecular Evolutionary Genetics Analysis version 6 (MEGA6, available online: http://www.megasoftware.net/) software was used to generate two separate phylogenetic trees to obtain classification based on rDNA sequence variation in the 11 Korean isolates, in the published *P. brassicae* isolates and in other cercozoa species [49]. The Maximum Likelihood method and Jukes-Cantor model, and also the Neighbour-Joining method and Maximum Composite Likelihood model, were used to generate phylogenetic trees. Another circular cladogram was constructed following the UPGMA method and Kimura 80 nucleotide distance measure method, using CLC Main Workbench version 7, taking all rDNA sequences. The secondary structure of variable regions of the large subunit was predicted using the mfold Web Server (available online: http://mfold.rna.albany.edu/?q=mfold). A%, T%, G% and C% in the variable region 2 of LSU was obtained using MEGA6 software. 

### 4.5. Probe and Primer Design and HRM Analysis

Probes were designed with 3′ phosphorylation for high resolution melting (HRM) and synthesized by Bioneer, Inc., Alamedia, CA, USA (Table 3). Forward and reverse primers covering the probe sequence were also synthesized by Oligo Macrogen, Seoul, Korea (Table 3). HRM was carried out using a real-time PCR (LightCycler 480, Roche Applied Science). The HRM PCR mix consisted of 1 μL genomic DNA at 5 ng·μL^−1^, 7.4 μL ultra-pure water, 0.1 and 0.5 μL forward and reverse primers, respectively, 1 μL probe, and 10 μL QuantiSpeed HRM Kit (PhileKorea, Deajeon, Korea). The polymerase chain reaction before the HRM was carried out with the following conditions: an initial pre-incubation at 95 °C for 10 min followed by 50 cycles of 95 °C for 20 s, annealing between 63 and 55 °C for 20 s under touchdown command, and 72 °C for 20 s. HRM data were recorded by five readings per °C at the final step after 60 s at 95 °C, 120 s at 40 °C, and 1 s at 83 °C. HRM curve analysis was conducted using LightCycler 96 software (Roche, Mannheim, Germany) at 75% discrimination for both delta Tm and curve shape with a 0.2 positive/negative threshold level. 

## 5. Conclusions

We sequenced the SSU, ITS1, ITS2, 5.8s and the major portion of the LSU of the rDNA of 11 Korean geographical isolates of *P. brassicae*. The nucleotide sequences of ITS1, 5.8s and ITS2 were generally conserved in the 11 isolates. However, a large deletion detected in the SSU between two groups of field isolates has evolutionary significance. Large sequence variations in two locations in the LSU in the 11 Korean geographical isolates and in the Japanese reference and European sequences indicate a potential variation in biosynthesis of the corresponding proteins. The variation observed in this work highlights the importance of studying the rDNA of *P. brassicae* isolates, as the isolates could be functionally different, especially in terms of disease development and severity. Four HRM markers revealed variation due to nucleotide polymorphism or InDels. Three of these markers discriminated the 11 Korean isolates into two groups. The HRM-based markers could be used to identify *P. brassicae* field isolates from different regions of Korea when associated intron 1 sequence is present.

## Figures and Tables

**Table 1:** List of primer sequences used for cloning and sequencing the rDNA of target region of *P. brassicae* (see Figure 1).

Name	Primer Forward (F) and Reverse (R)	Position	Product Size
CR_rDNA 1	F: GGTTGATCCTGCCAGTAGTC	6–774	768
	R: GAAGCGATTCGGGCATAGAG		
CR_rDNA 2	F: CCTATGCTAATCTCGTGGCG	728–2182	1454
	R: TAAGAAGTCACGGACCTAGG		
CR_rDNA 3	F: TTATGCCTATGCCTTCCCGG	2164–3192	1028
	R: GAAACACGCAGCTGGAGTCGC		
CR_rDNA 4	F: TCCGTAGGTGAACCTGCGGA	3080–4142	1062
	R: CCCATTCTGACCTAGGCCAA		
CR_rDNA 5	F: TTGGCCTAGGTCAGAATGGG	4110–5606	1496
	R: ATGCGGTTATGAGTACGACCA		
CR_rDNA 6	F: CAGGTTCATATTCCTGAACC	5346–6981	1635
	R: CGTTCAAATCAAGTCGTCTAC		

**Table 2:** GenBank accession numbers of the sequenced clones.

Different Geographic Isolates	Accession No.	Sequence Length (bp)	Sequence Encoding
Gangneung1	KX430457	6931	Partial ribosomal RNA, both small and large subunits
Yeoncheon	KX430458	6937	
Daejon	KX430459	6935	
Haenam2	KX430460	6927	
Seosan	KX430461	6923	
Pyeongchang	KX430462	6932	
Gangneung2	KX430463	6931	
Haenam1	KX430464	6935	
Hoengseong	KX430465	6934	
Geumsan	KX430466	6923	
Goesan	KX430467	6929	

**Table 3:** Position and sequences of designed probes and primers used for high resolution melting to detect melting temperature variation between isolates after high resolution melting.

Probe Name	Position	Probe Sequence	Nucleotide Variation	Froward (F) and Reverse (R) Primers	Position	Product Size (bp)
Probe_594	594	CAGTGTCTGTTT**A**CTGTTGGCGCCA	A/G	F: TTAAACCTATTATCGAGGATCC R: ACGAGATTAGCATAGGATTATC	530–742	212
Probe_629	629–632	GGCTTCTCTTTT**---**AAAGAAGTTATGC	**---**/ATT			
Probe_661	662	TCGAGCAGCCCA**T**TCTAGTTGTGGG	T/A			
Probe_856	857	TCGAAAGAATGC**C**GATCGACTGGAG	C/T	F: GAAGCGATTCGGGCATAGAGGCC R: CGCTATTGGAGCTGGTATTACC	756–982	226
Probe_903	904–912	CGGGGGTGTGTG**----**TTTGATGGAGGAA	**----**/CGCG			
Probe_1364	1366	AGCATTCACCAA**-**GGATGTCCTCTTT	**-**/A	F: GAAGTAATGATTGATAGGGATAGT R: CGACCGCCAATCCCTAGTCGAC	1262–1465	203
Probe_3564	3561–3562	ACACACACACACA_TCAAAGATCTCA	**--**/CA	F: GCTGCGGCATAGCTTGAACGAA R: CGGTTTGAGCTCTTCCCGCTTC	3448–3685	237
Probe_5516	5545	TCCGA**C**GGA**C**AGA**C**TTATTCGCA	C/G, C/A, C/T	F: TCAGGTTCATATTCCTGAACCA R: ACCTGATGCGGTTATGAGTACG	5316–5574	258
Probe_6479	6479	ATGAATACAAAC**T**GTGAAAGCATGGC	T/C	F: CGATCTACGCGAGAGACAAAGTC R: GCAACGTCGCTATGAACGCTTG	6315–6594	279
Probe_6759	6759	AGTAATCCAATT**C**AGTACGAGAGGA	C/T	F: ATCATTGCCTCGCAGCAGAGGCC R: AGAGGCGTTCAGCCGTAATCCT	6624–6860	236

## Supplementary Materials

Supplementary materials can be found at www.mdpi.com/1422-0067/18/1/84/s1.

## Abbreviations

rDNA: Ribosomal DNA; ITS: Internal transcribed spacer; PCR: Polymerase chain reaction; SSU: small subunit; LSU: large subunit; IGS: intergenic spacer; HRM: high resolution melting; SNP: single-nucleotide polymorphism.
