# Supplementary material for: Detection of Ribosomal DNA Sequence Polymorphisms in the Protist Plasmodiophora brassicae for the Identification of Geographical Isolates
Source: Int J Mol Sci. 2017 Jan 4;18(1):84. doi: 10.3390/ijms18010084 (PMC5297718; doi:10.3390/ijms18010084)
Supplement: Supplementary file 1 [file ijms-18-00084-s001.pdf]

Supplementary Materials: Detection of Ribosomal DNA Sequence Polymorphisms in the Protist *Plasmodiophora brassicae* for the Identification of Geographical Isolates

Rawnak Laila, Arif Hasan Khan Robin, Kiwoung Yang, Gyung Ja Choi, Jong-In Park and Ill-Sup Nou

Table S1. Nucleotide sequence variation among 11 *P. brassicae* isolates in "region 2" of large subunit (LSU) as in Figure 4.

Isolate	T	C	A	G	GC (%)	Total
AB526843	23.2	22.4	25.9	28.5	50.9	509
Gangneung 1	22.0	23.5	24.3	30.2	53.7	510
Yeoncheon	22.0	23.5	24.3	30.2	53.7	510
Daejon	22.0	23.5	24.3	30.2	53.7	510
Haenam 2	23.8	21.7	25.8	28.7	50.4	512
Seosan	23.8	21.7	25.8	28.7	50.4	512
Phyongchang	23.5	21.7	26.5	28.3	50	506
Gangneung 2	22.0	23.5	24.3	30.2	53.7	510
Haenam 1	21.8	24.1	24.1	30.0	54.1	510
Hoengseong	21.8	24.1	24.1	30.0	54.1	510
Geumsan	23.6	21.8	26.5	28.1	49.9	509
Goesan	23.6	21.8	26.5	28.1	49.9	509
Average	22.8	22.7	25.3	29.2	51.9	510

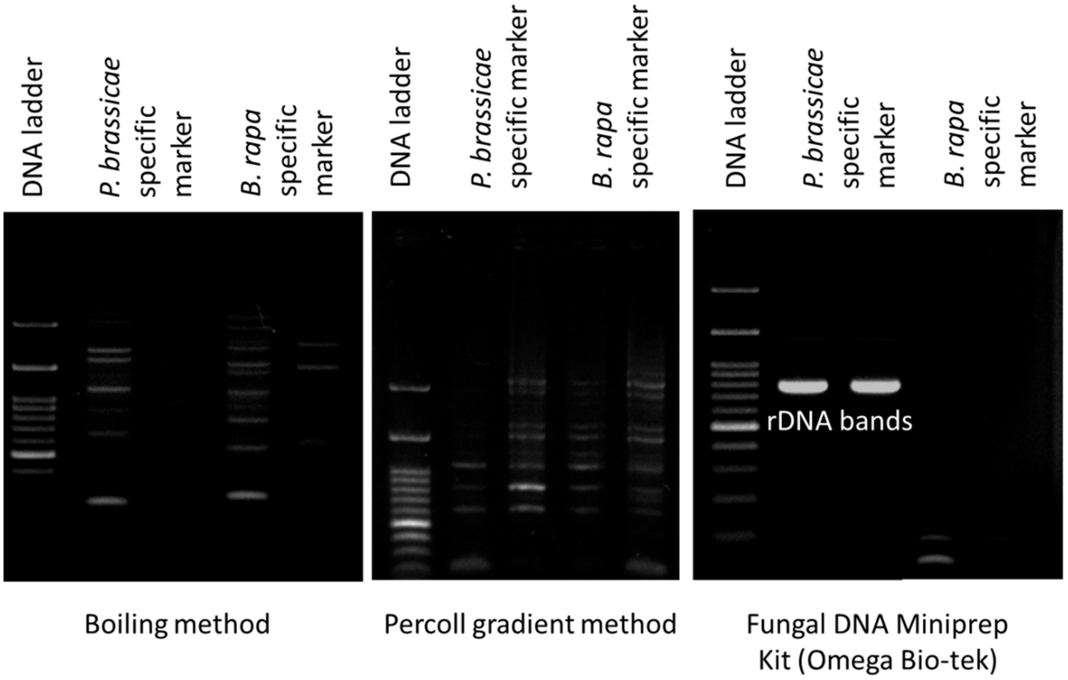

Figure S1. *P. brassicae*-specific markers and *B. rapa*-specific markers show rDNA bands of *P. brassicae* and DNA bands of Chinese cabbage.

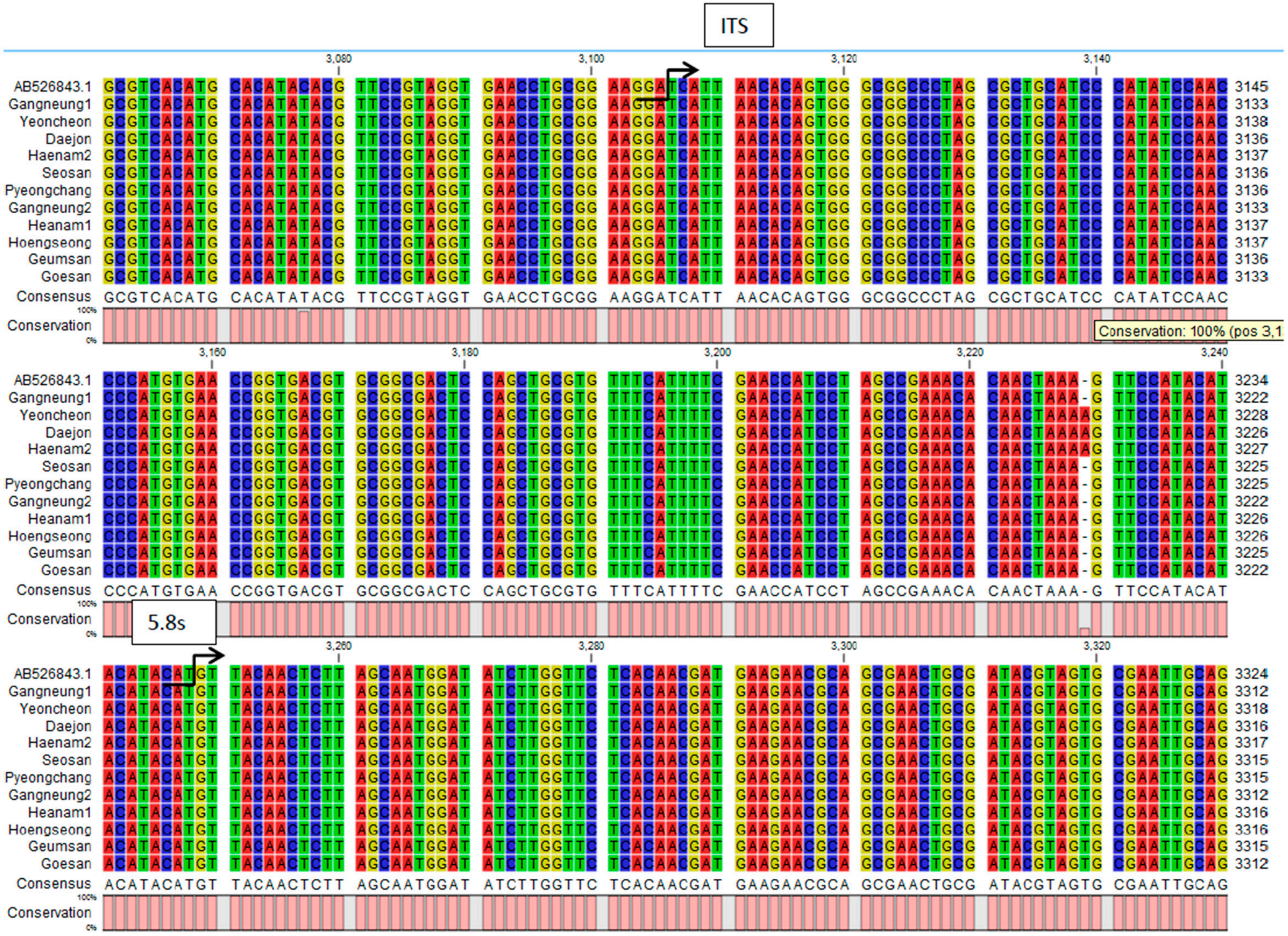

Figure S2. *Cont.*

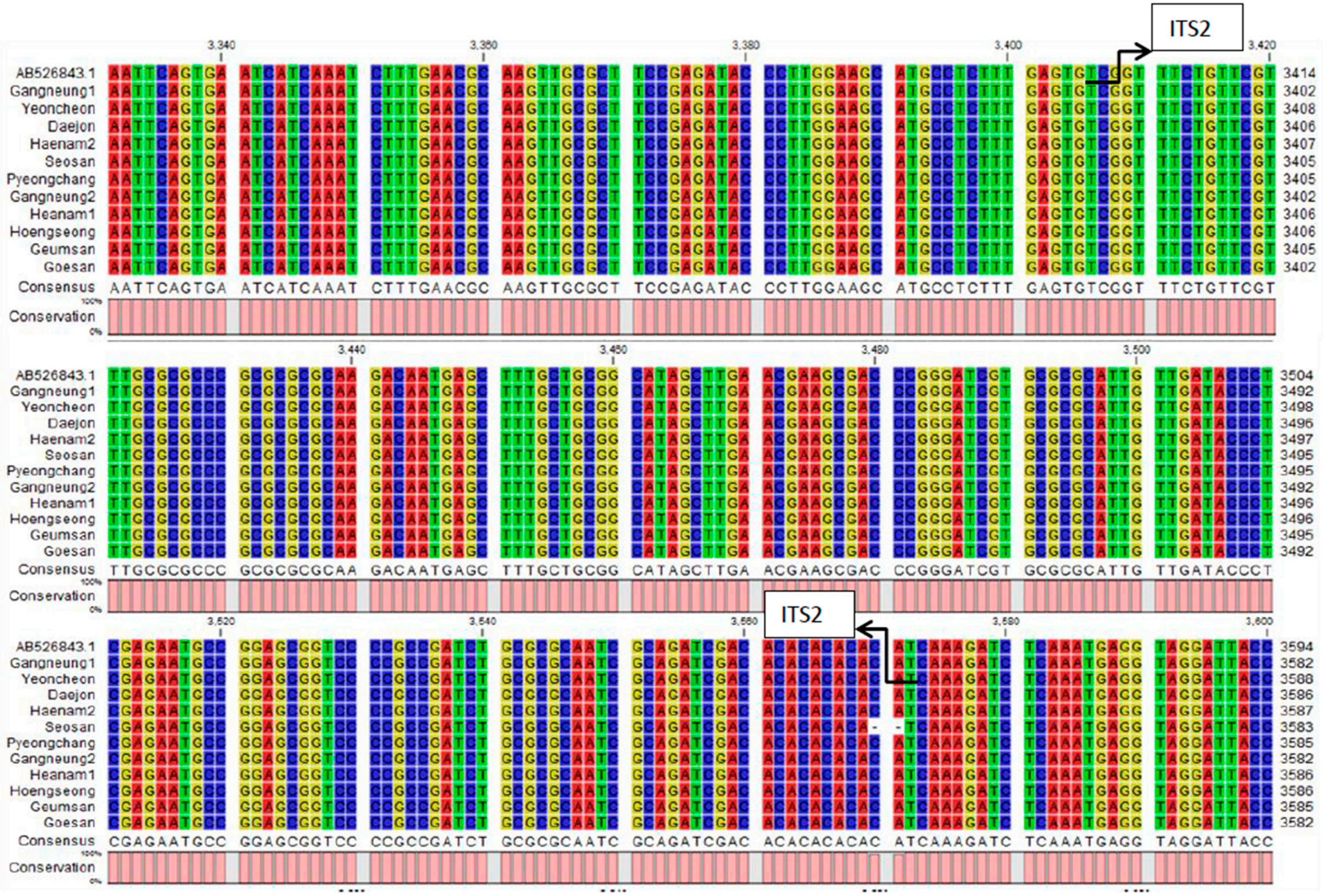

Figure S2. Nucleotide Sequence of ITS1 (3106–3247 bp), 5.8s (3248–3408) and ITS2 (3409–3570) region. Blue, yellow, red and green colors represent C, G, A and T residues.

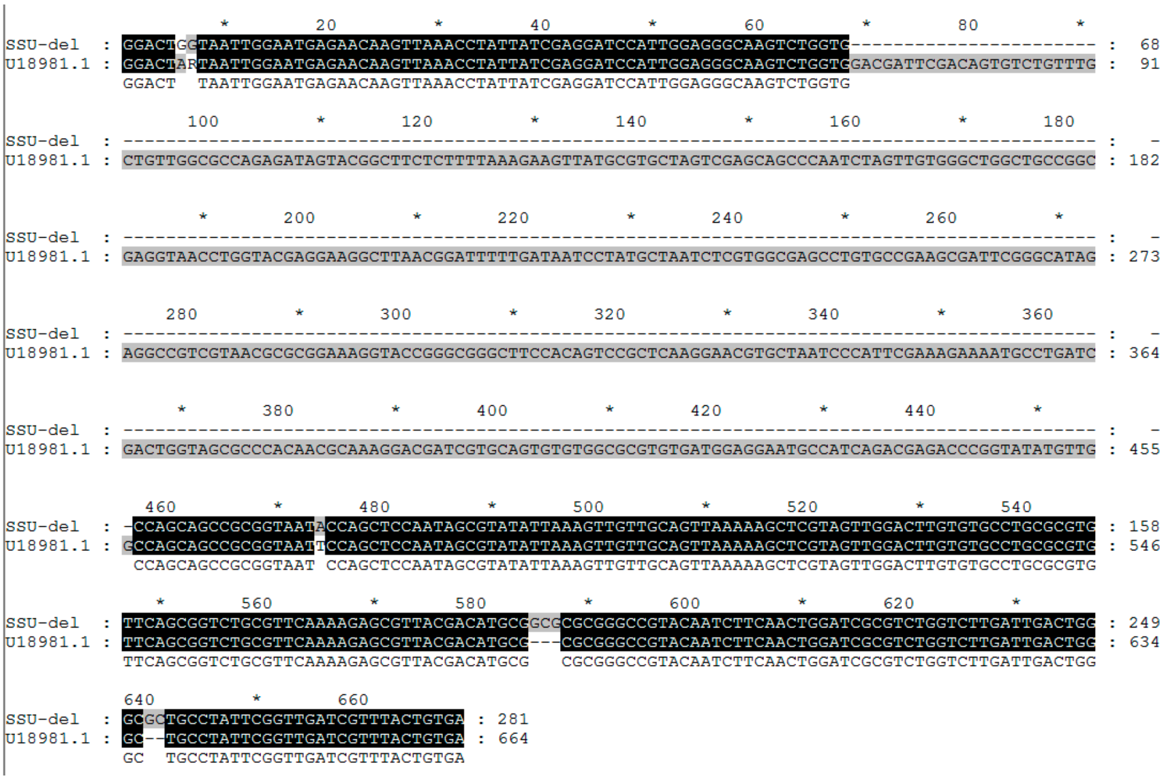

Figure S3. SSU sequence comparison between two groups of Korean *P. brassicae* isolates showing a 388-bp deletion.

Sequences 4425-4594

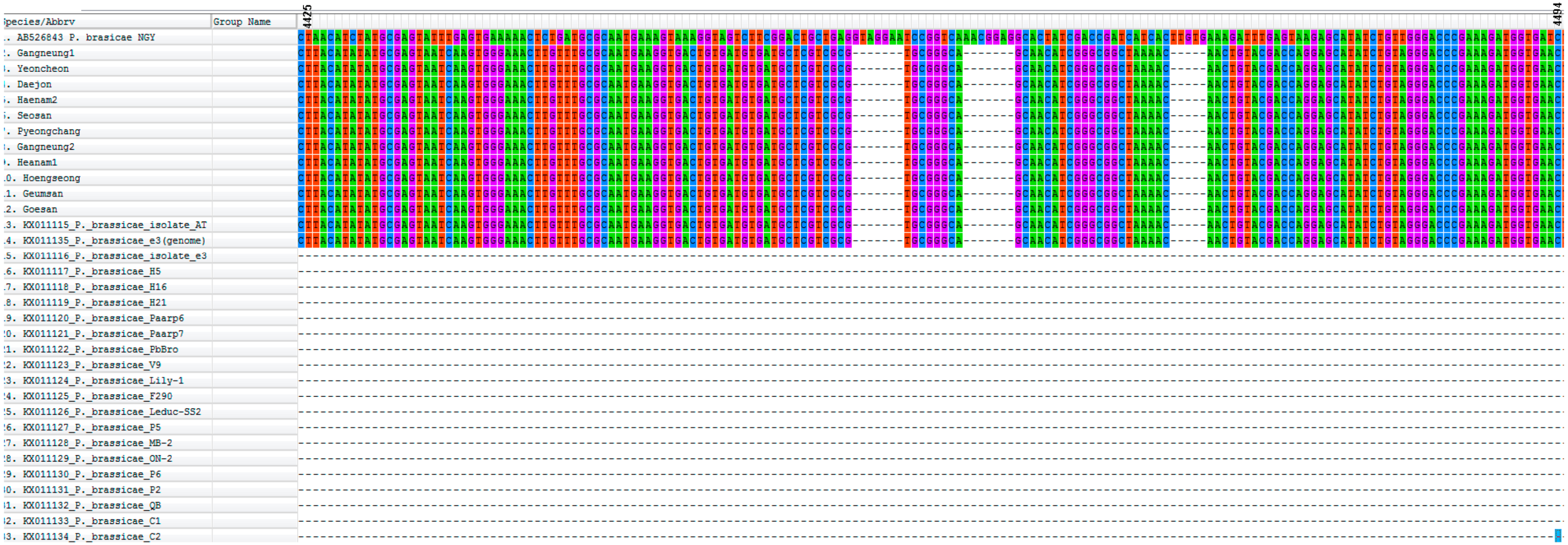

Figure S4. *Cont.*

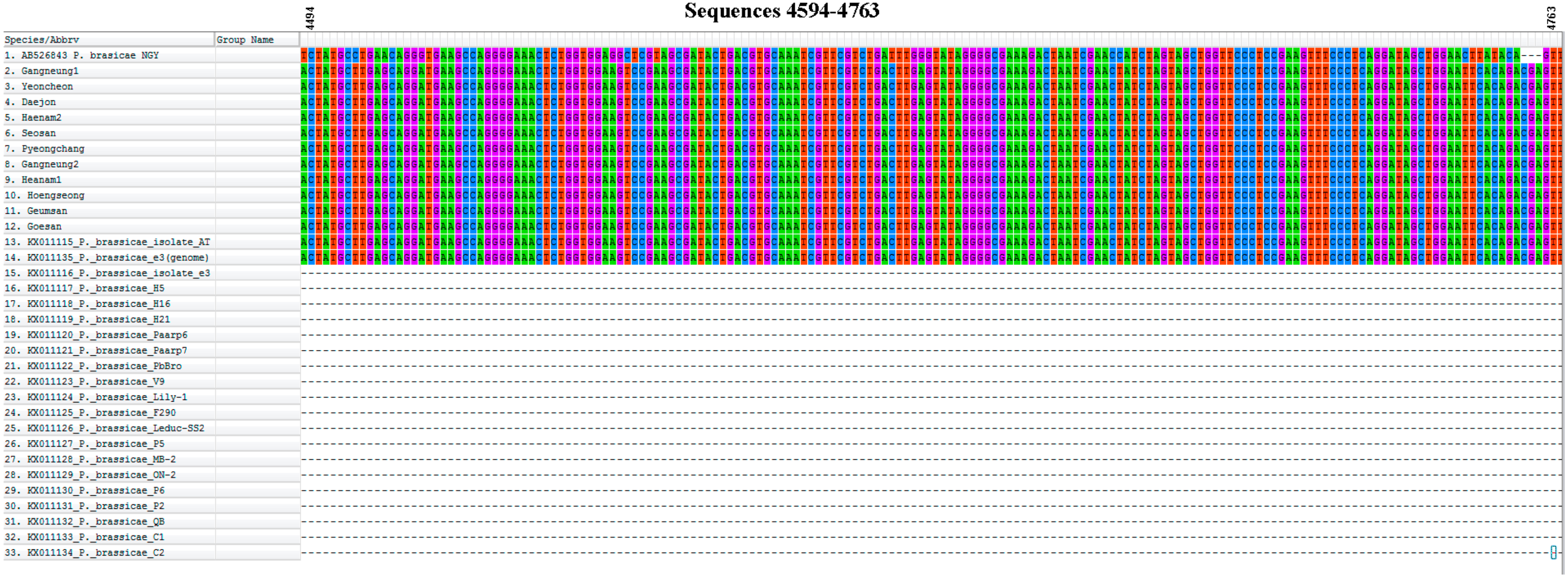

Figure S4. *Cont.*

Sequences 4763-4932

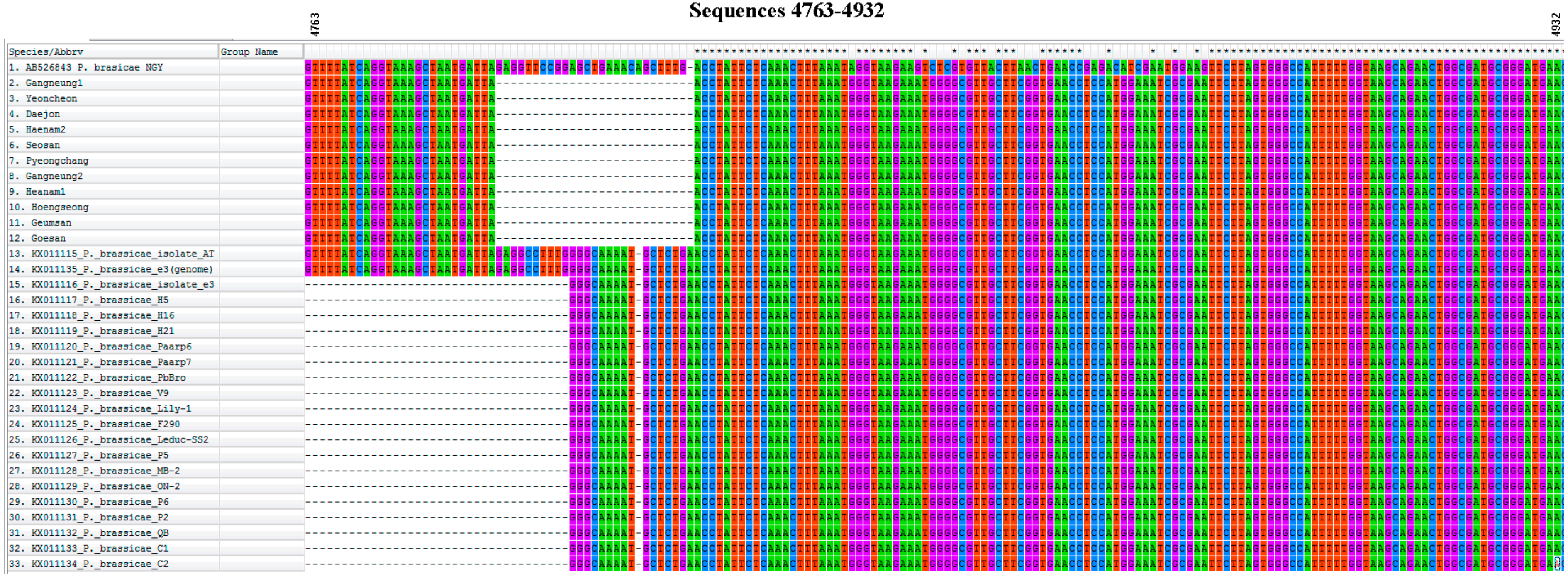

Figure S4. *Cont.*

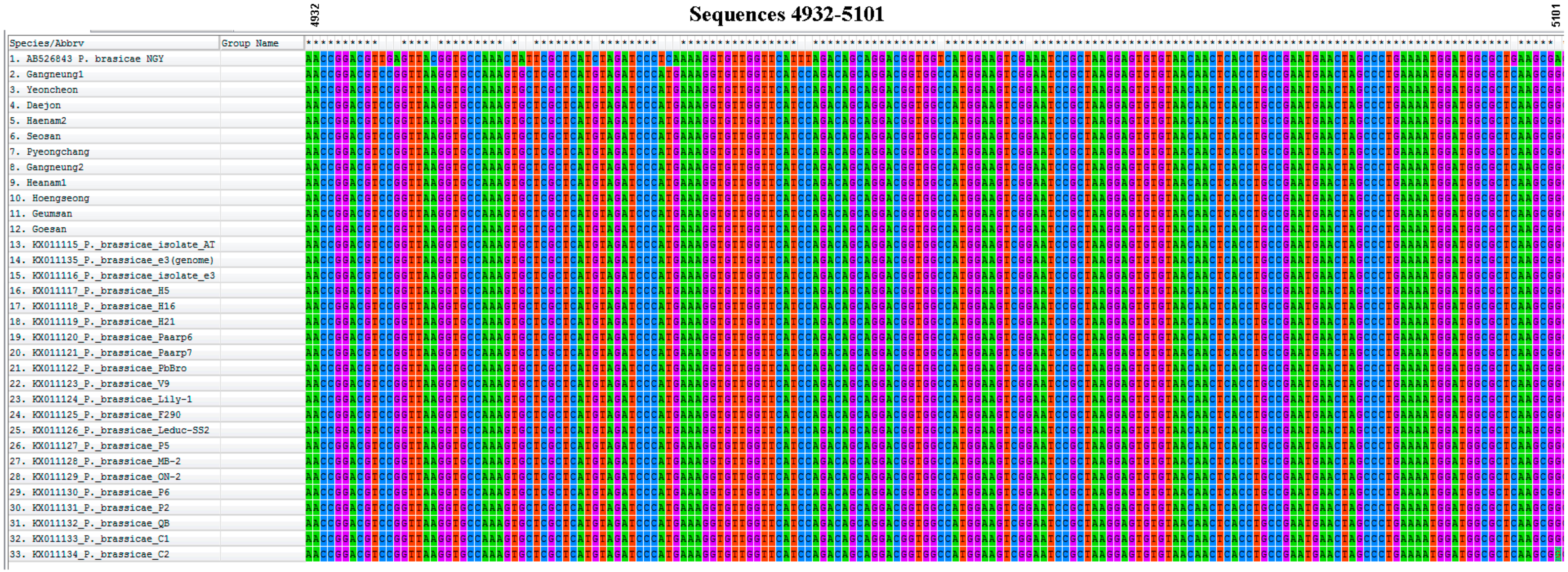

Figure S4. *Cont.*

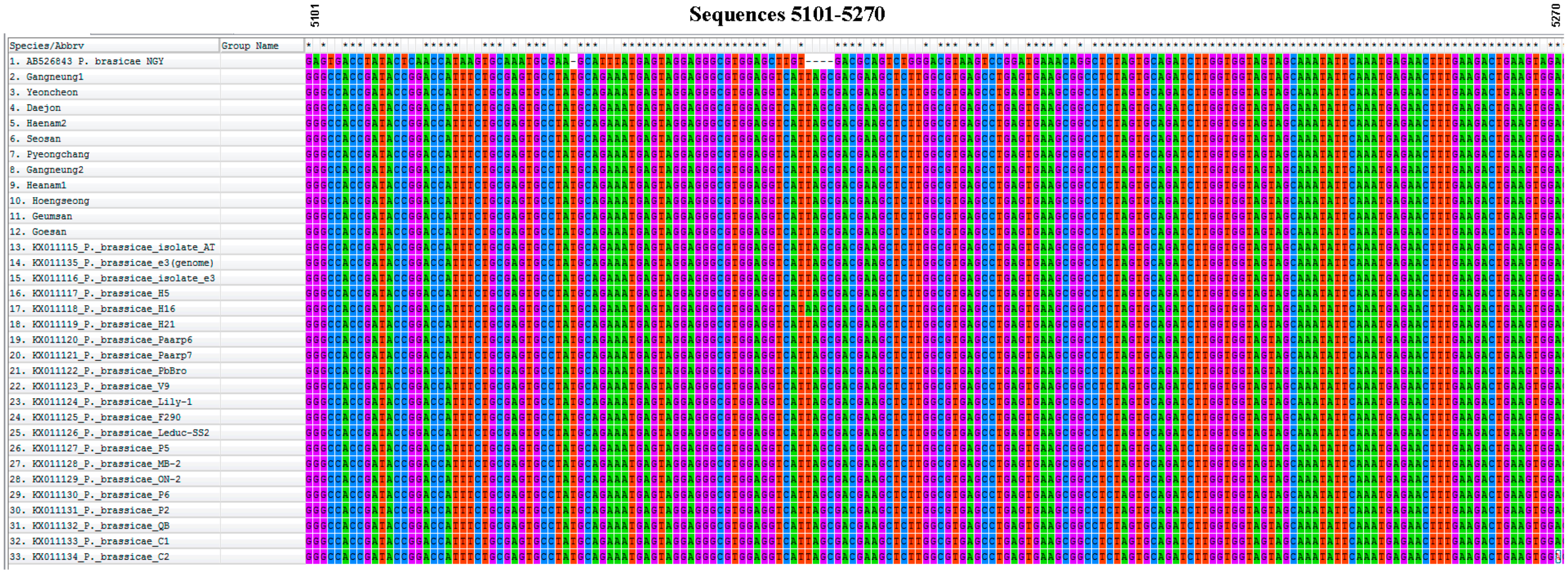

Figure S4. *Cont.*

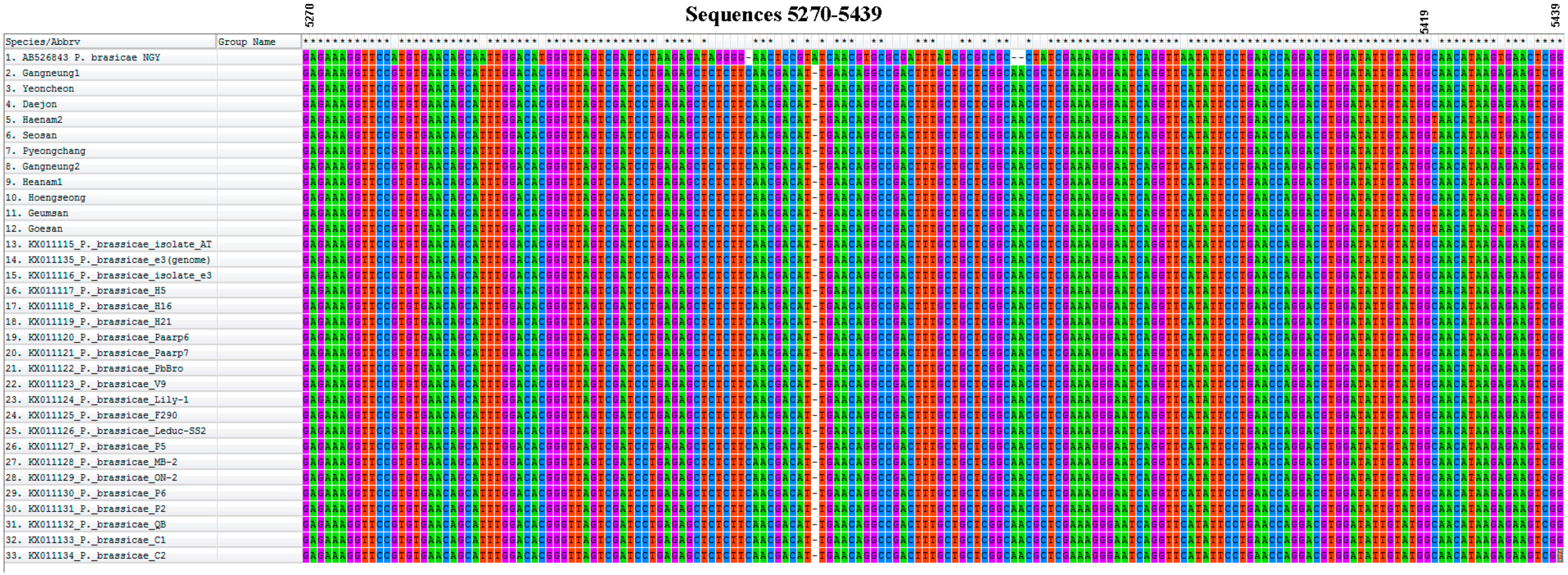

Figure S4. *Cont.*

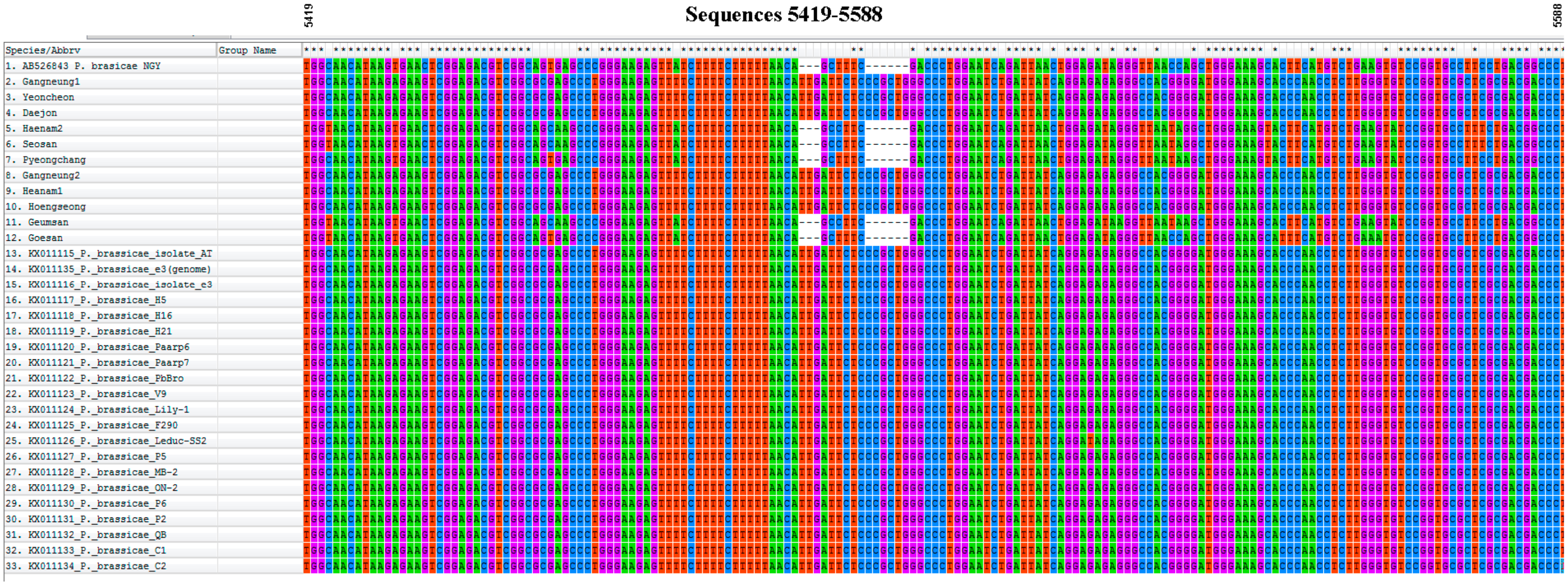

Figure S4. *Cont.*

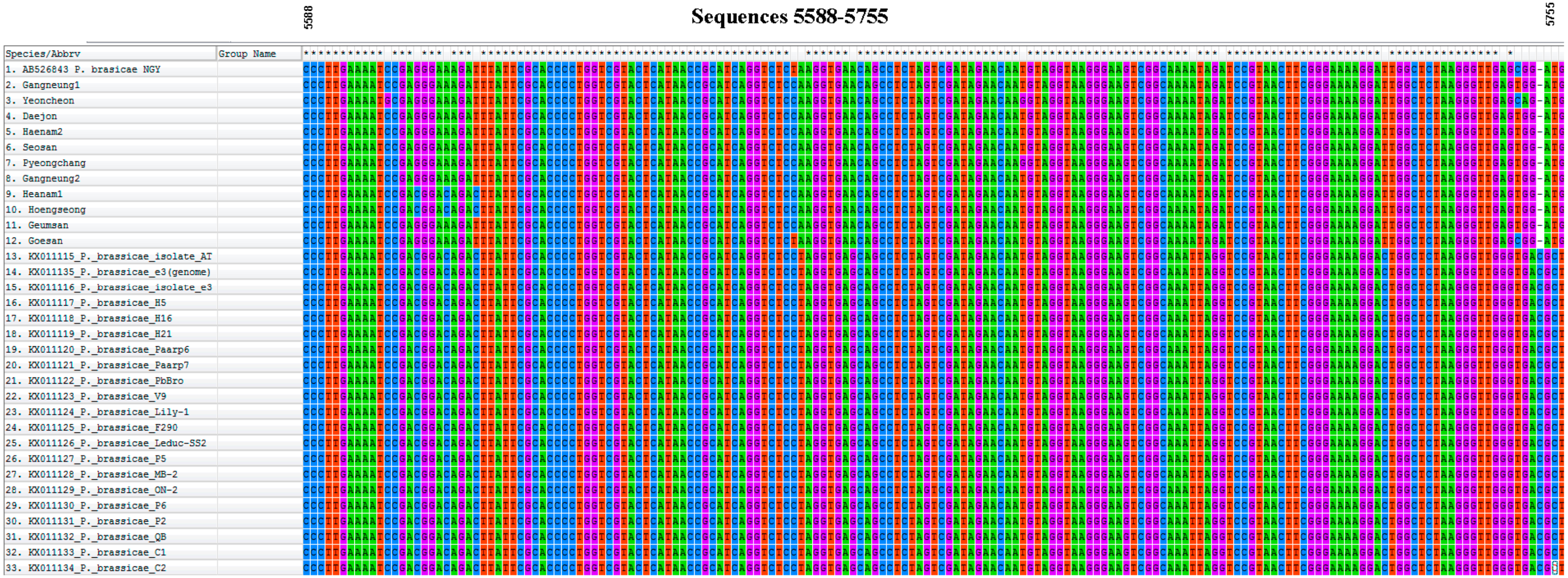

Figure S4. *Cont.*

Sequences 5755-5924

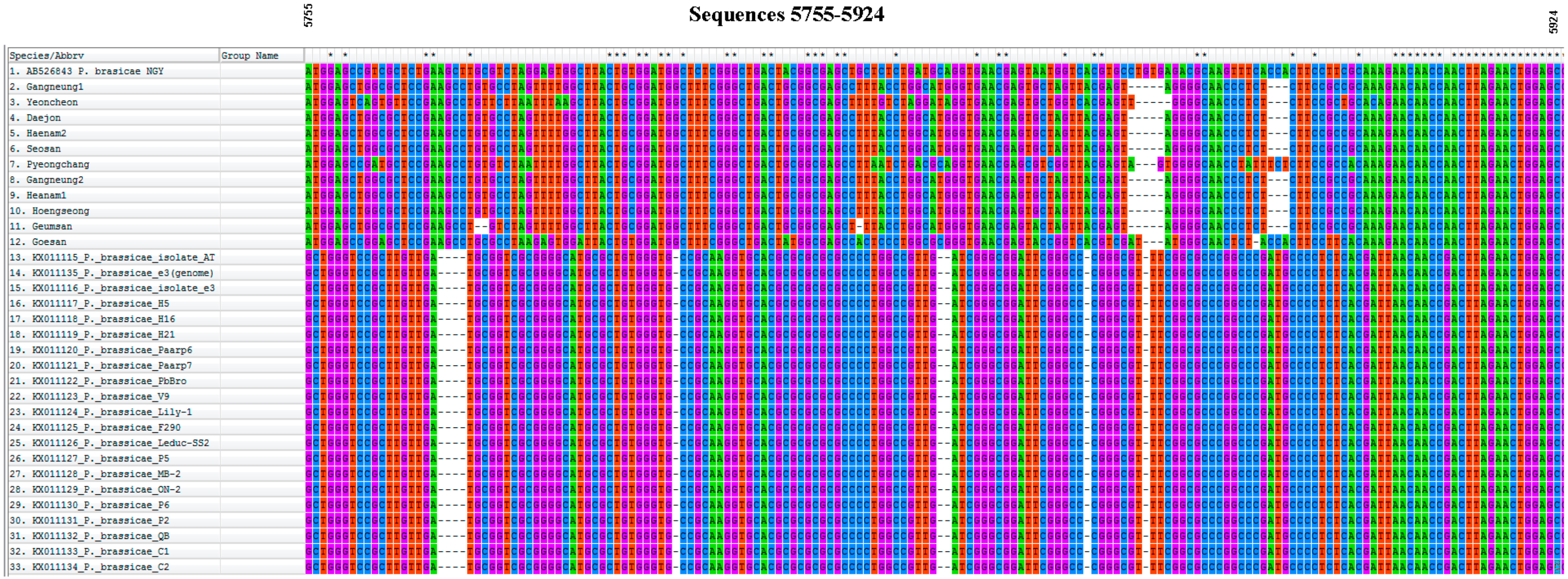

Figure S4. *Cont.*

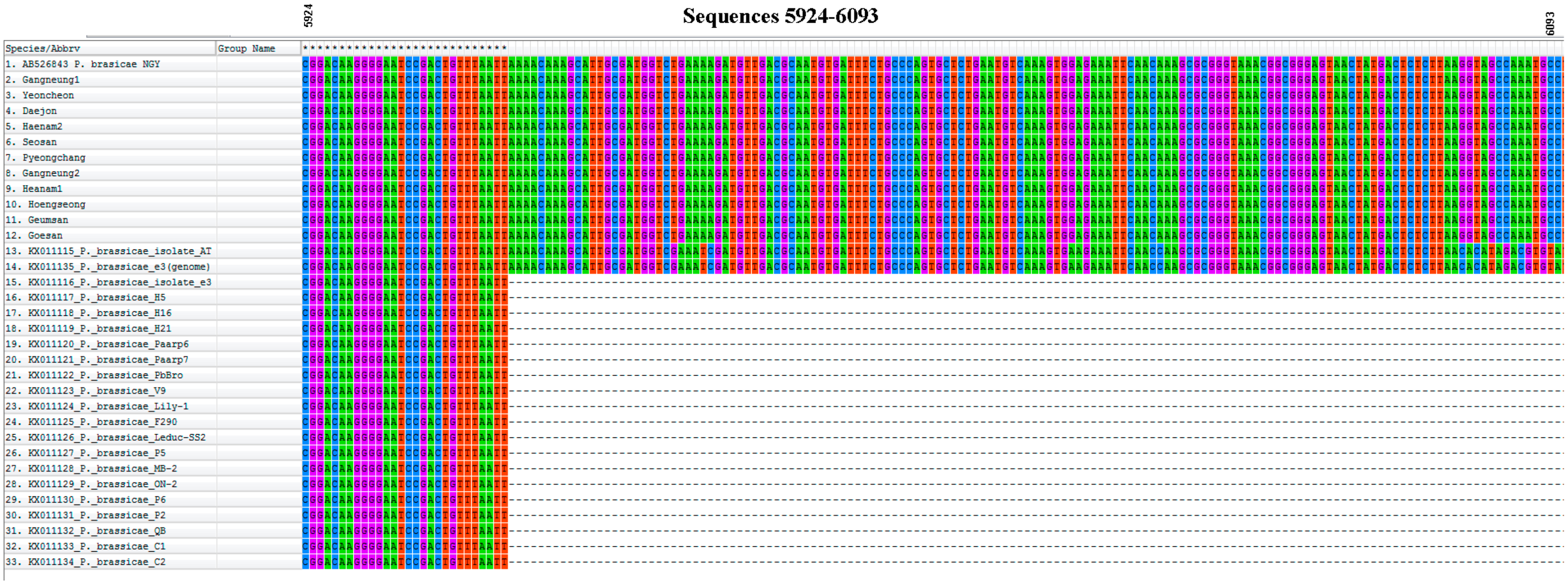

Figure S4. *Cont.*

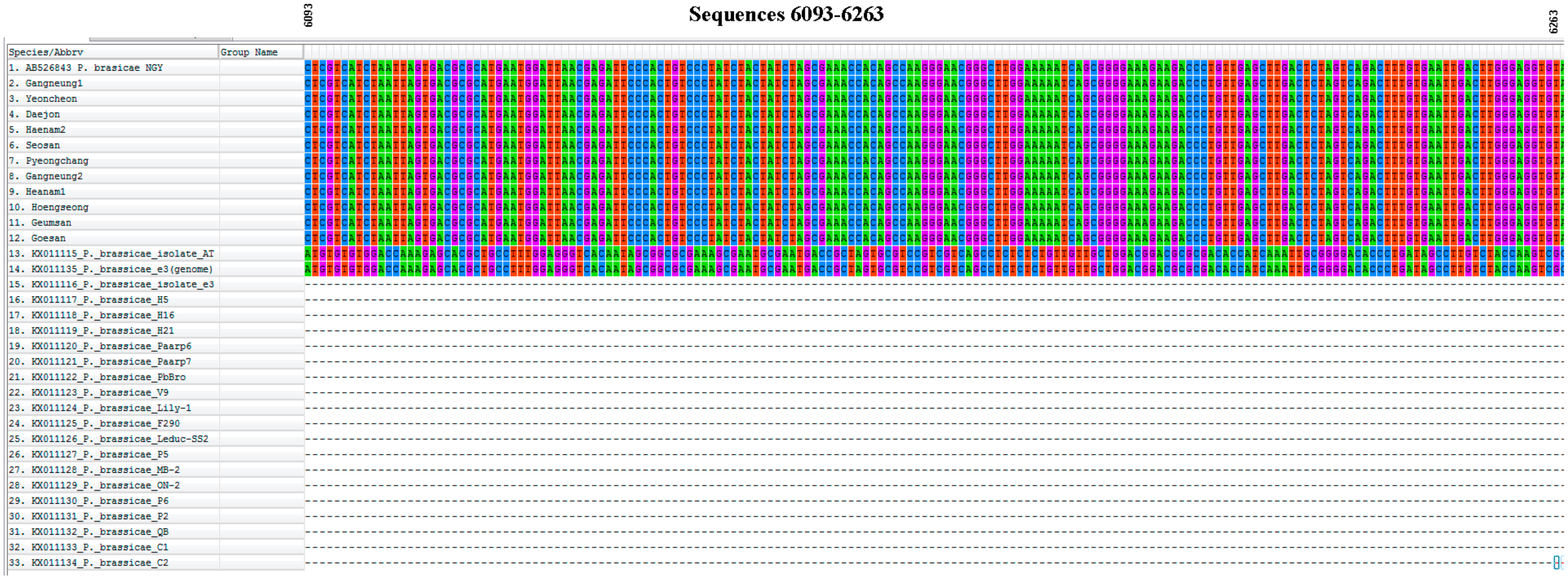

Figure S4. *Cont.*

Sequences 6263-6433

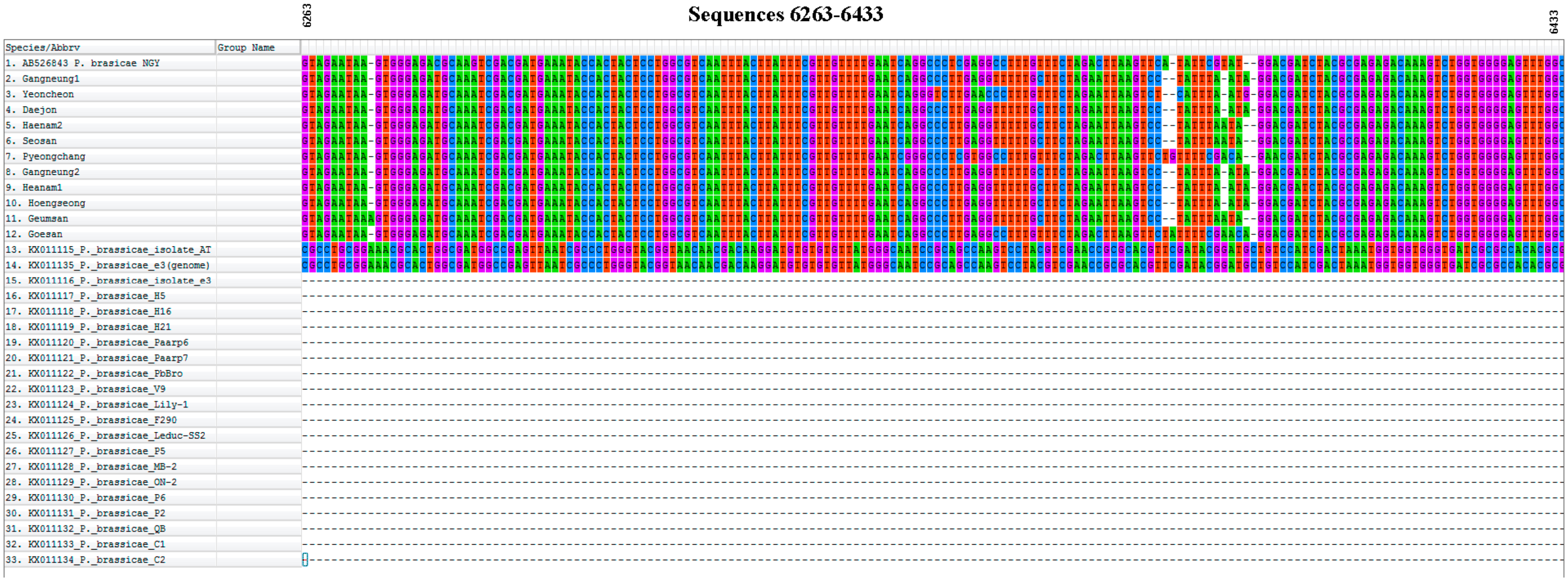

Figure S4. *Cont.*

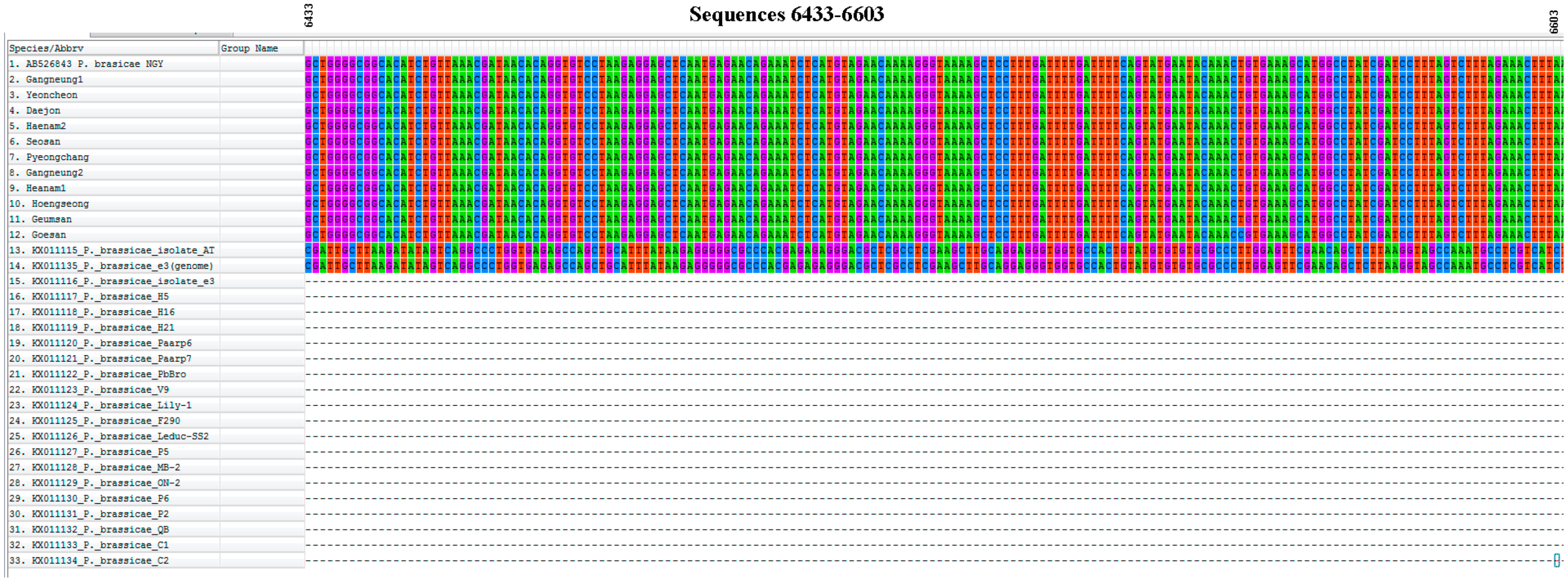

Figure S4. *Cont.*

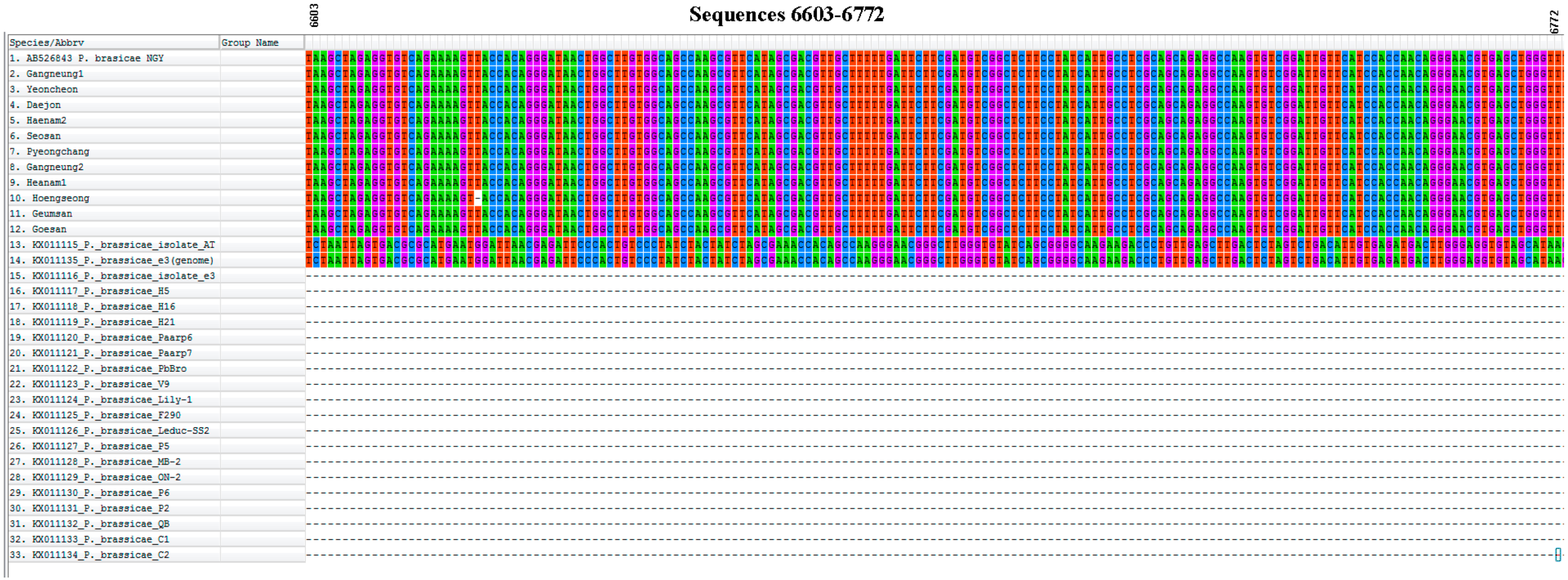

Figure S4. *Cont.*

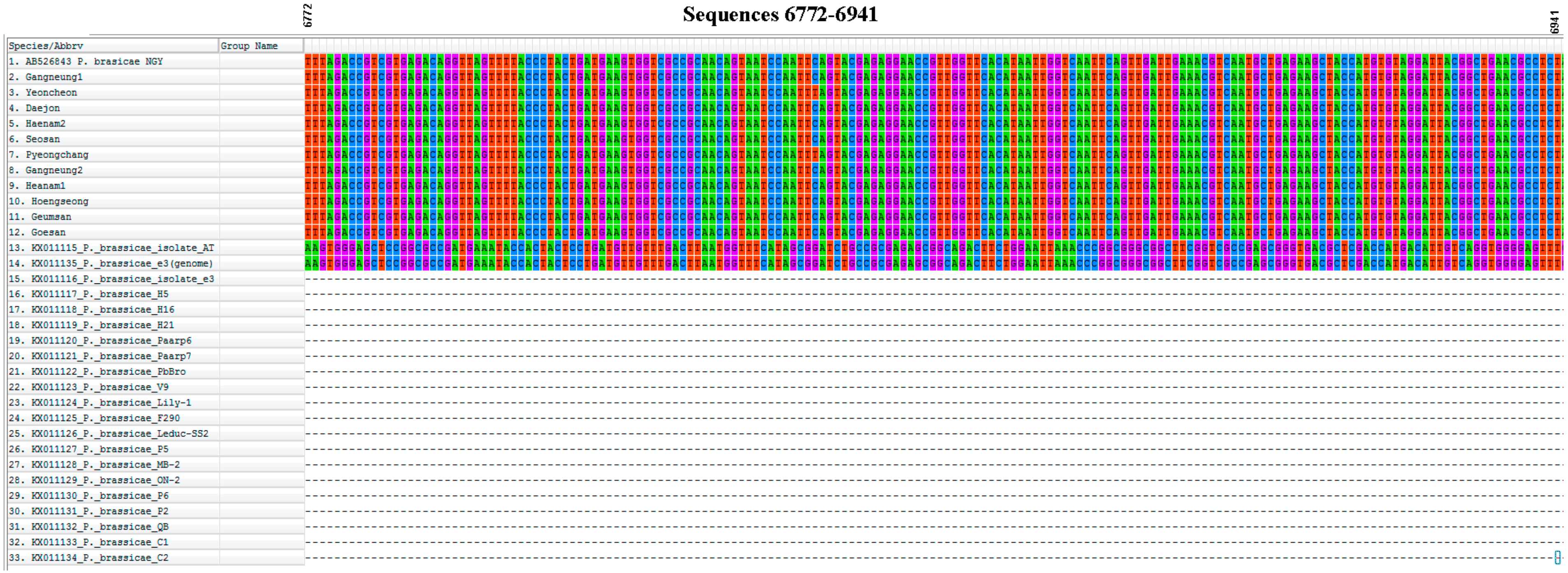

Figure S4. *Cont.*

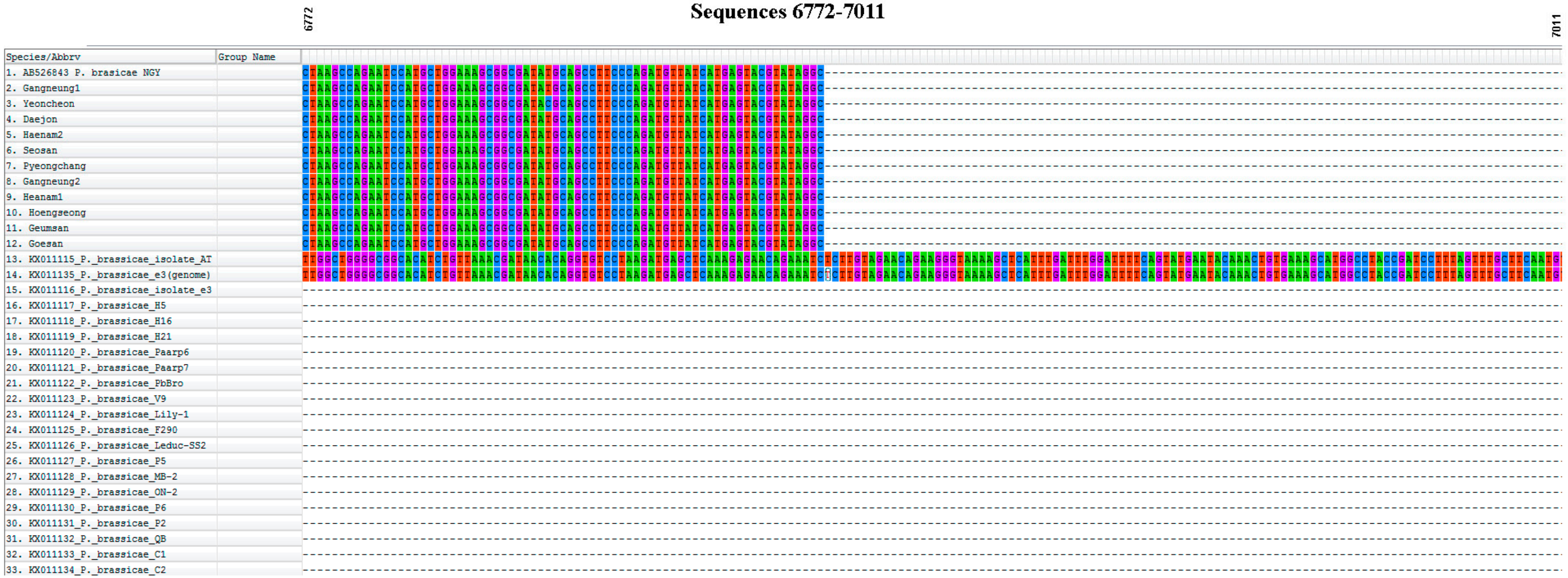

Figures S4. LSU sequence variation in Korean *P. brassicae* isolates compared to reference sequences. Green, red, purple and blue colors represent A, T, G and C residues.

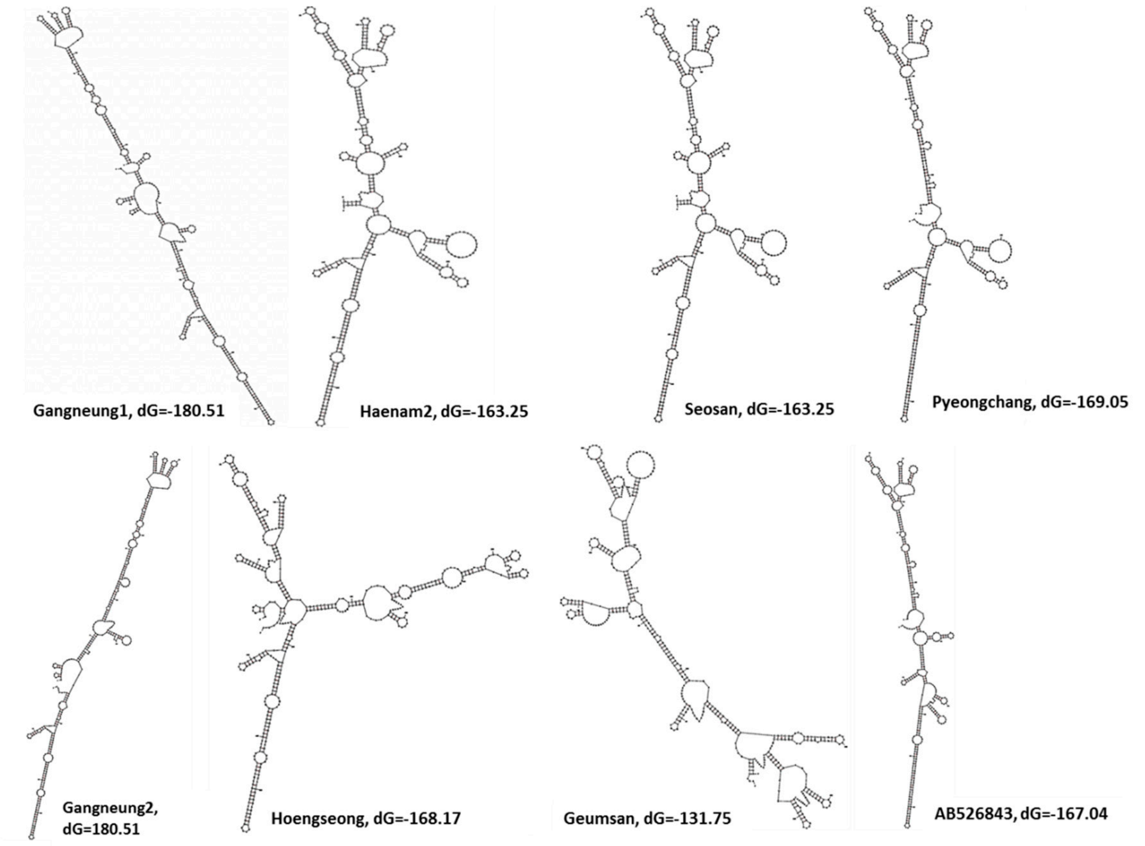

Figure S5. Predicted secondary structures. dG estimates free-energy in kcal·mol^{-1}.

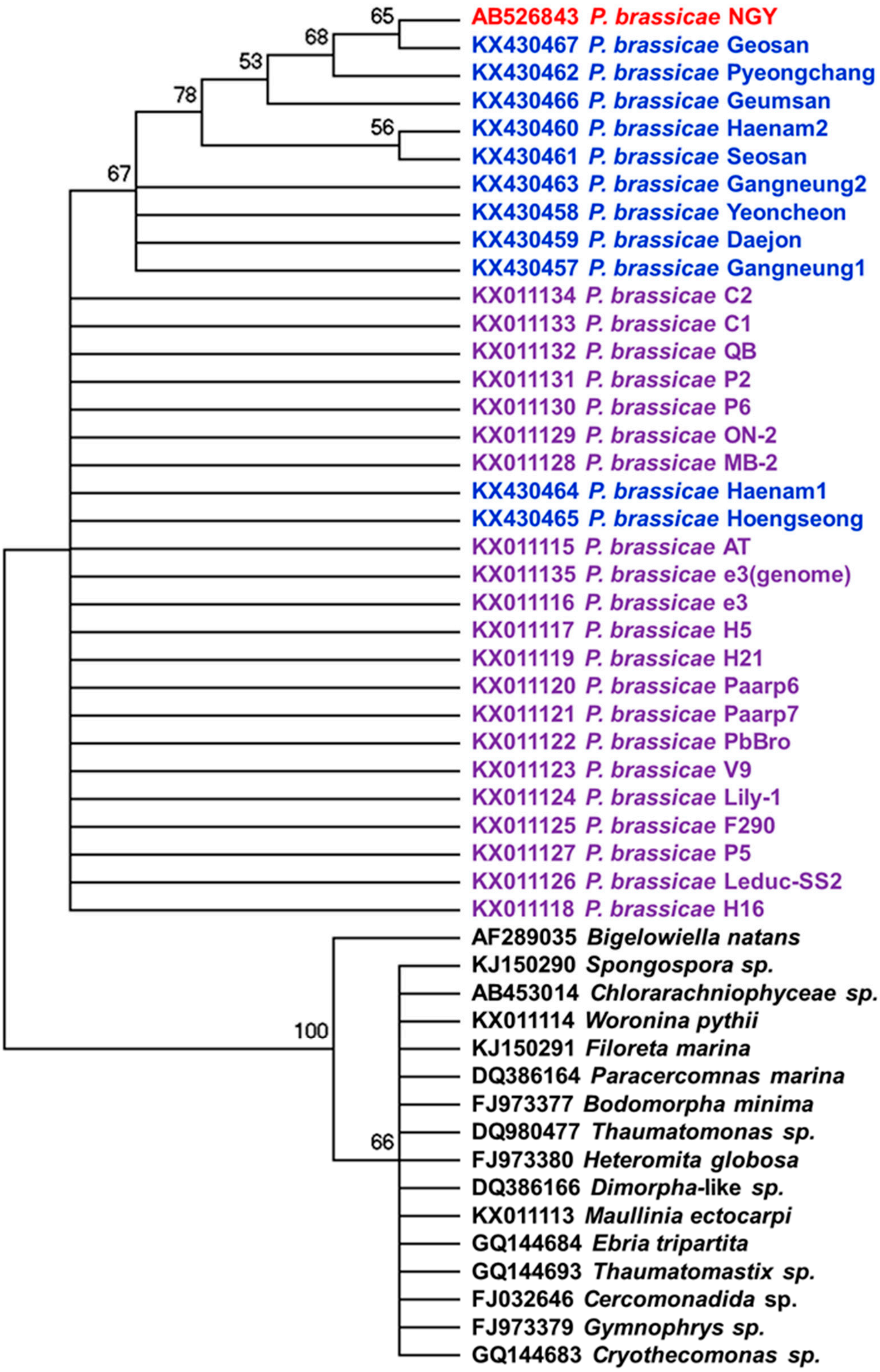

(**A**)

Figure S6. *Cont.*

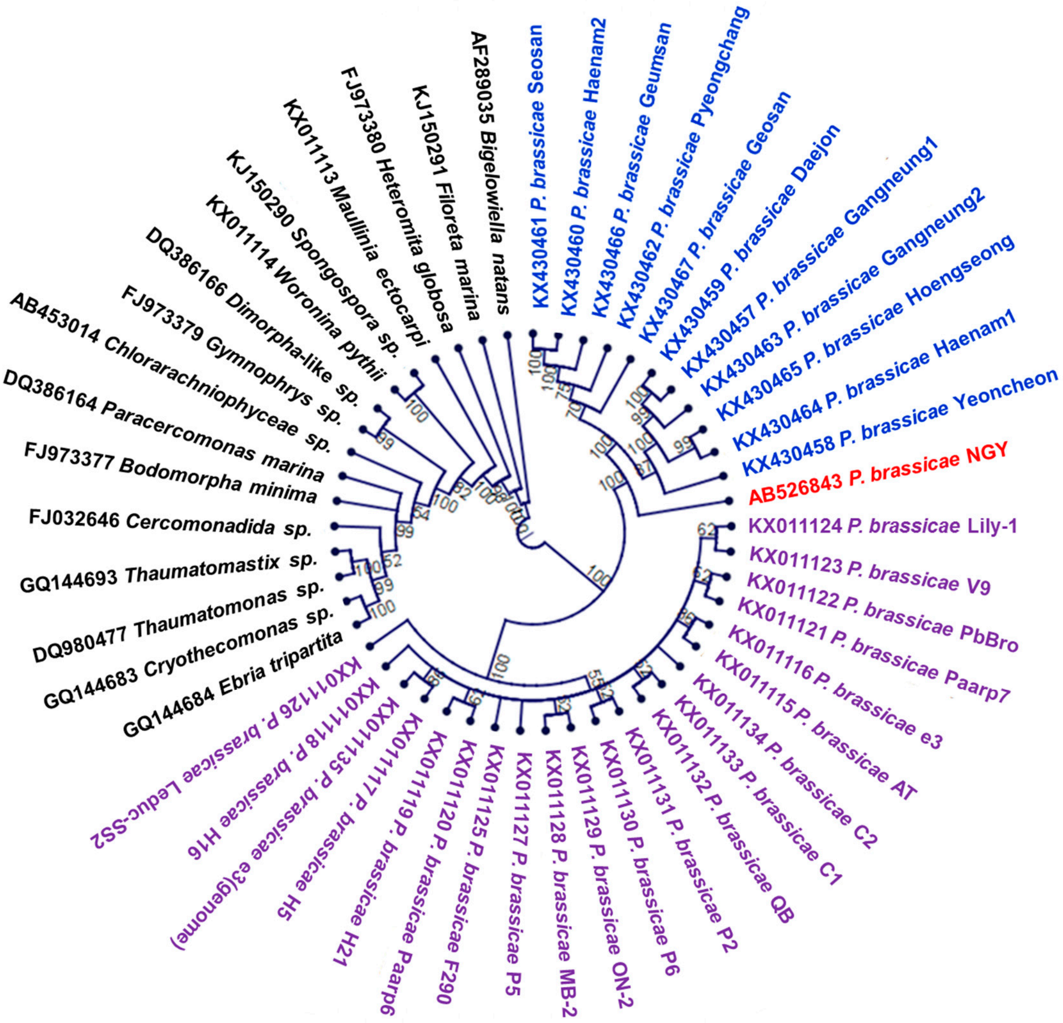

(**B**)

Figure S6. Phylogenetic classification of *P. brassicae* isolates and other cercoza species based on variations in the nucleotide sequences of rDNA. Purple, red and blue colors indicate *P. brassicae* rDNA sequences obtained from Schwelm et al. [32], Niwa et al. [31] and this study. Black colors indicate rDNA sequences from cercozoa species. Complete rDNA, if available or LSU sequences were used. (**A**) Phylogenetic tree constructed following the Neighbour-Joining method and Maximum Composite Likelihood model in Mega6.06 software and (**B**) Circular cladogram constructed following the UPGMA method and Kimura 80 nucleotide distance measure method in CLC Main Workbench version 7.

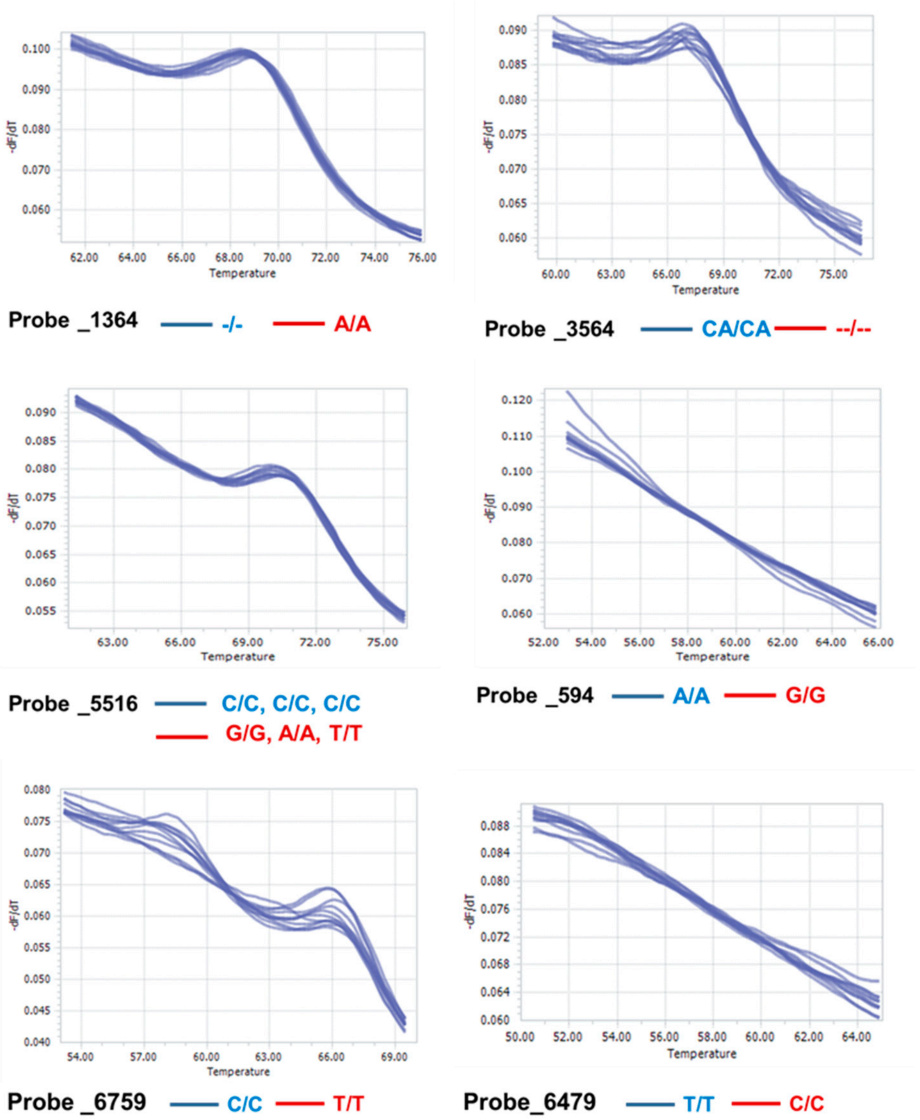

Figure S7. Absence of functional polymorphism (absence of red lines) between Korean *P. brassicae* isolates. Melting temperatures are shown for probes (single, oligo and multiple-single nucleotide polymorphism and InDel) designed at the LSU after high-resolution melting analysis in a Roche light cycler.

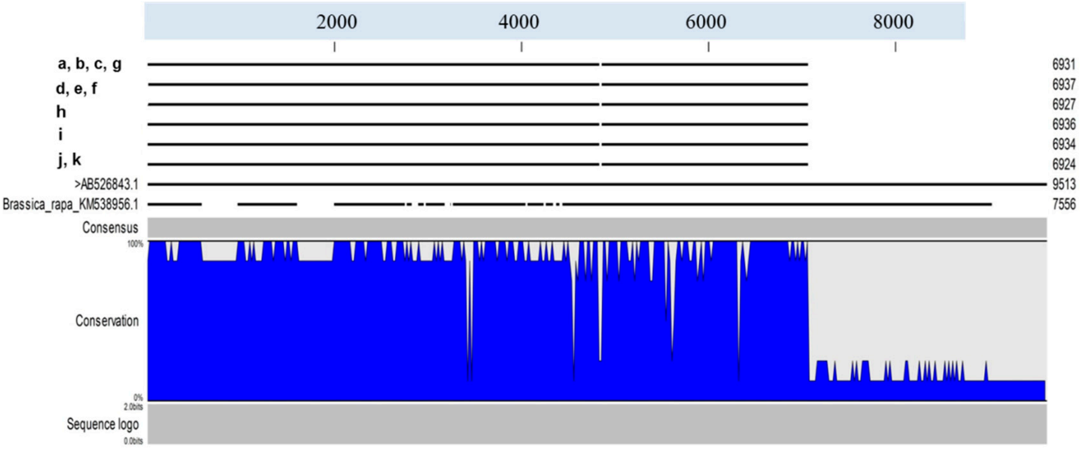

Figure S8. Variation in annotated sequences between *Plasmodiophora brassicae* and *Brassica rapa* larger subunit (LSU) rDNA sequences.

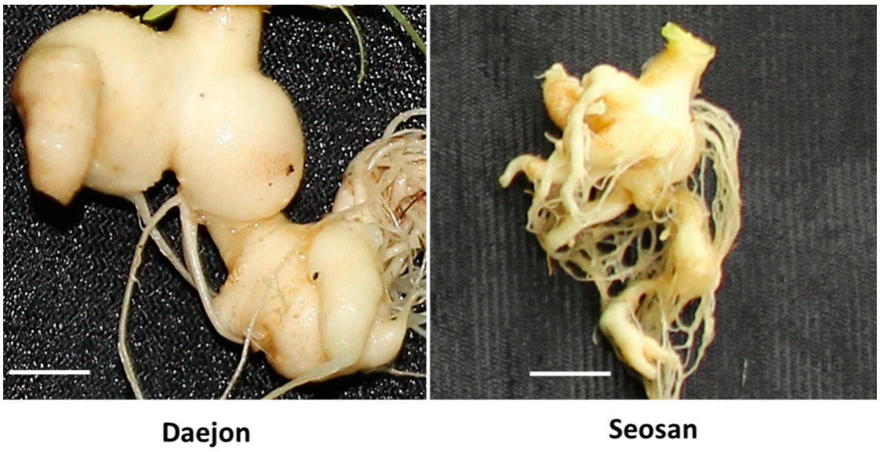

Figure S9. Variation in gall size produced by Korean *P. brassicae* isolates Daejon and Seosan, 28 days after inoculation. Scale = 0.5 cm.
